# Senescence‐Inducing Therapy Sequential NIR‐II Mild Photothermal/Senolytic Therapy of Triple Negative Breast Cancer

**DOI:** 10.1002/advs.202507248

**Published:** 2025-08-13

**Authors:** Liya Yu, Yehui Kang, Mengdie Yu, Yuejia Ding, Yang Chen, Qingyuan Wu, Yu Cai, Huiyu Liu, Zhenye Lv

**Affiliations:** ^1^ General Surgery, Cancer Center, Department of Breast Surgery, Zhejiang Provincial People's Hospital (Affiliated People's Hospital) Hangzhou Medical College Hangzhou Zhejiang 310014 P. R. China; ^2^ Beijing Advanced Innovation Center for Soft Matter Science and Engineering, State Key Laboratory of Organic‐Inorganic Composites, Beijing Laboratory of Biomedical Materials, Bionanomaterials & Translational Engineering Laboratory, Beijing Key Laboratory of Bioprocess Beijing Laboratory of Biomedical Materials Beijing University of Chemical Technology Beijing 100029 P. R. China

**Keywords:** CDK4/6 inhibitor, mild PTT, senescence‐inducing therapy, senolytics, triple negative breast cancer

## Abstract

Targeting the limited therapeutic options and frequent drug resistance in triple negative breast cancer (TNBC) remains a critical clinical challenge. Here, a novel sequential theranostic mild strategy leveraging cellular senescence to enhance mild photothermal therapy (PTT) and promote TNBC eradication is presented. Palbociclib, a clinically‐approved CDK4/6 inhibitor, is used to induce senescence in a murine TNBC model, resulting in both tumor growth arrest and increased heat sensitivity due to HSP70 downregulation. Capitalizing on this vulnerability, an NIR‐II theranostic nanoplatform (IQ NPs) composed of the photothermal agent IR1061 and the senolytic quercetin (Qu), fabricated via nanoprecipitation is developed. IQ NPs demonstrated enhanced mild PTT efficacy, further potentiated by Qu‐mediated HSP70 suppression and augmented senolysis. This findings highlight the potential of exploiting senescence‐inducing vulnerabilities to amplify mild PTT efficacy, providing a promising new therapeutic avenue for this aggressive and recalcitrant malignancy.

## Graphical abstract

Targeting triple‐negative breast cancer remains challenging due to limited therapies and drug resistance. A sequential theranostic strategy combining palbociclib‐induced senescence (enhancing heat sensitivity via HSP70 downregulation) with an NIR‐II nanoplatform (IQ NPs) for mild photothermal therapy (PTT) and quercetin‐mediated senolysis is developed. This approach amplifies PTT efficacy, offering a promising therapeutic avenue for TNBC.

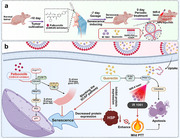

## 1 Introduction

Triple‐negative breast cancer (TNBC), characterized by the absence of estrogen receptor (ER), progesterone receptor (PR), and human epidermal growth factor receptor 2 (HER2) expression,^[^ ^1^, ^2^, ^3^ ^]^ poses a significant therapeutic challenge due to its aggressive biological behavior and intrinsic resistance to endocrine therapy and HER2‐targeted agents.^[^ ^4^ ^]^ Currently, the standard of care for TNBC relies heavily on cytotoxic chemotherapy (particularly platinum‐ and taxane‐based regimens) combined with surgical resection and radiotherapy.^[^ ^5^, ^6^, ^7^ ^]^ However, these approaches demonstrate substantial limitations. First, surgical intervention often fails to achieve complete tumor eradication in locally advanced cases, with high rates of recurrence and distant metastasis;^[^ ^8^ ^]^ Furthermore, conventional chemotherapy exhibits significant off‐target toxicity and lacks tumor specificity, leading to severe side effects that compromise patients' quality of life;^[^ ^9^ ^]^ Lastly, radiotherapy, while useful for local control, does not address systemic disease and may cause long‐term tissue damage.^[^ ^10^ ^]^ Although emerging neoadjuvant strategies, including neoadjuvant endocrine therapy, immunotherapy and hyperthermia, have been explored to downstage tumors preoperatively, clinical outcomes remain suboptimal, with less than 30% of patients achieving pathological complete response and no demonstrated survival benefit in most trials.^[^ ^11^ ^]^ Moreover, the marked intertumoral heterogeneity and rapid chemoresistance development in TNBC further complicate treatment selection and efficacy. These critical gaps in current therapies underscore the urgent need for novel, mechanism‐driven treatment paradigms that can overcome therapeutic resistance while minimizing systemic toxicity—particularly for younger patients who frequently present with this aggressive subtype.^[^ ^12^ ^]^

Photothermal therapy (PTT) has emerged as an innovative tumor ablation technique that utilizes photothermal agents to convert NIR light into localized cytotoxic heat, offering a minimally invasive treatment modality with significant clinical potential for therapy‐resistant tumors.^[^ ^13^, ^14^, ^15^ ^]^ However, two major limitations hinder its broader application: restricted tissue penetration depth, which can be partially addressed using NIR‐II window agents such as IR1061,^[^ ^16^, ^17^ ^]^ and thermoresistance mediated by heat shock protein (HSP) upregulation, particularly HSP70, under mild PTT (≤45 °C).^[^ ^18^ ^]^

Cellular senescence, a defense mechanism characterized by stable cell cycle arrest with maintained metabolic activity, plays a dual role in tumor biology, influencing cancer initiation, progression and treatment response.^[^ ^19^, ^20^ ^]^ While CDK4/6 inhibitors (palbociclib, ribociclib, and abemaciclib) have been FDA‐approved for HR+/HER2‐ breast cancer treatment,^[^ ^21^, ^22^ ^]^ their potential in triple‐negative breast cancer warrants further investigation. Notably, senescent tumor cells exhibit downregulated HSP70 expression,^[^ ^23^, ^24^, ^25^, ^26^ ^]^ suggesting a potential therapeutic synergy with mild PTT. However, the biological consequences of senescence are complex while initially tumor‐suppressive through cell cycle arrest and immune‐mediated clearance via senescence‐associated secretory phenotype (SASP),^[^ ^27^, ^28^ ^]^ persistent senescent cells may paradoxically promote malignancy through chronic inflammation and epithelial‐mesenchymal transition.^[^ ^29^ ^]^ The development of senolytic agents, first proposed by James et al. in 2015,^[^ ^30^ ^]^ provides a strategic solution to selectively eliminate senescent cells. Among these, quercetin (Qu) demonstrates particular promise due to its dual mechanisms: inhibiting HSP70 expression through suppression of heat shock transcription factor (HSF) phosphorylation while concurrently acting as an effective senolytic agent.

**Scheme 1:**
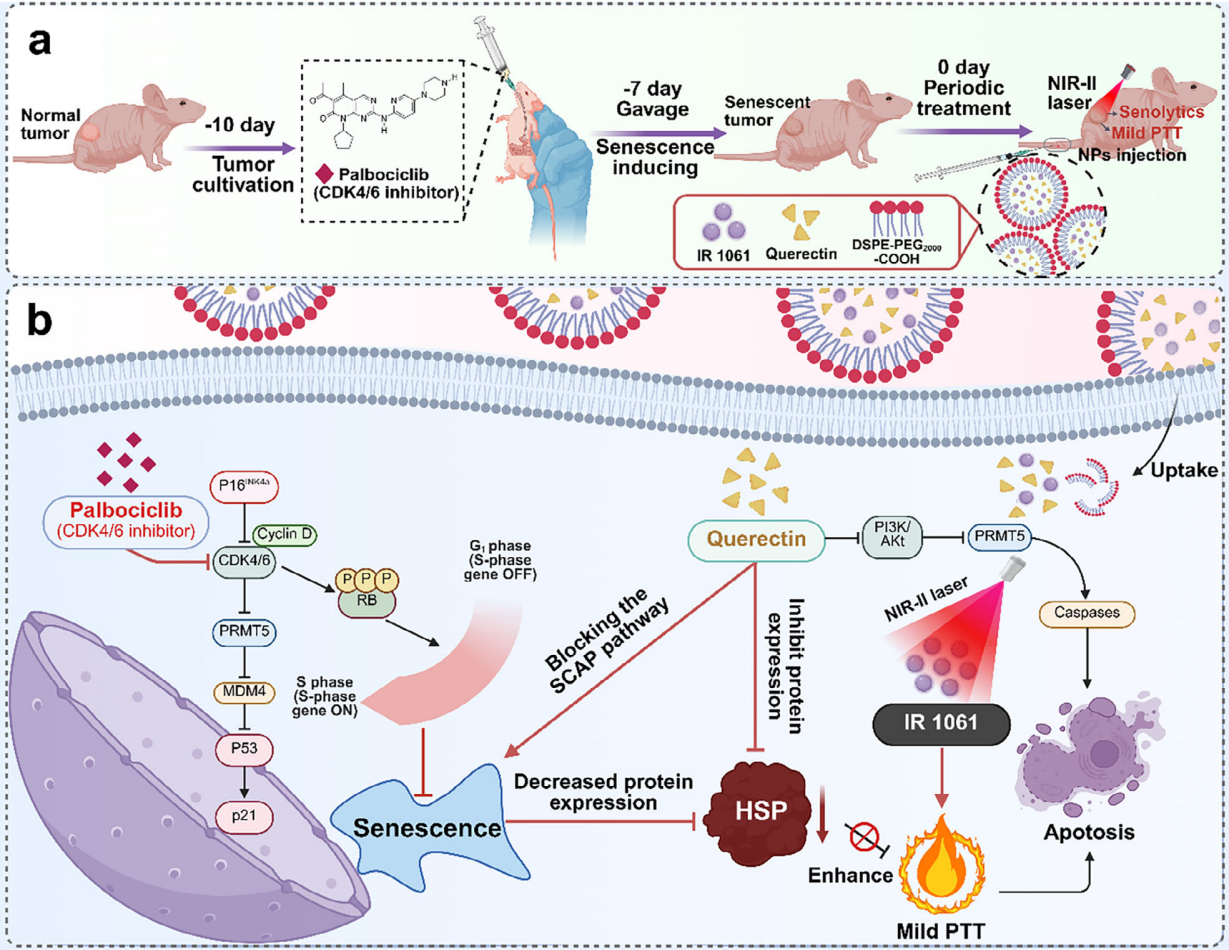
Schematic diagram of synergistic anti‐tumor mild PTT and senolytics based on senescent tumor. a) Schematic diagram of the in vivo combination therapy process. b) Treatment mechanism in the senescent tumor cells.

This study develops an innovative multimodal therapeutic approach that strategically combines NIR‐II‐mediated mild PTT with cellular senescence induction and targeted senolysis for enhanced TNBC treatment (Scheme **1**). This combination approach integrating mild PTT to induce tumor‐selective senescence by using CDK4/6 inhibitors like palbociclib.^[^ ^31^ ^]^ leveraging the intrinsic HSP70 downregulation in senescent cells, and employing Qu for both HSP70 inhibition and senescent cell clearance represents a rationally designed therapeutic strategy that capitalizes on the complementary advantages of each modality while mitigating their individual limitations, potentially yielding optimized treatment outcomes.^[^ ^32^, ^33^ ^]^

## 2 Results and Discussion

### 2.1 In Vitro Induced Senescence Validation

**Figure 1:**
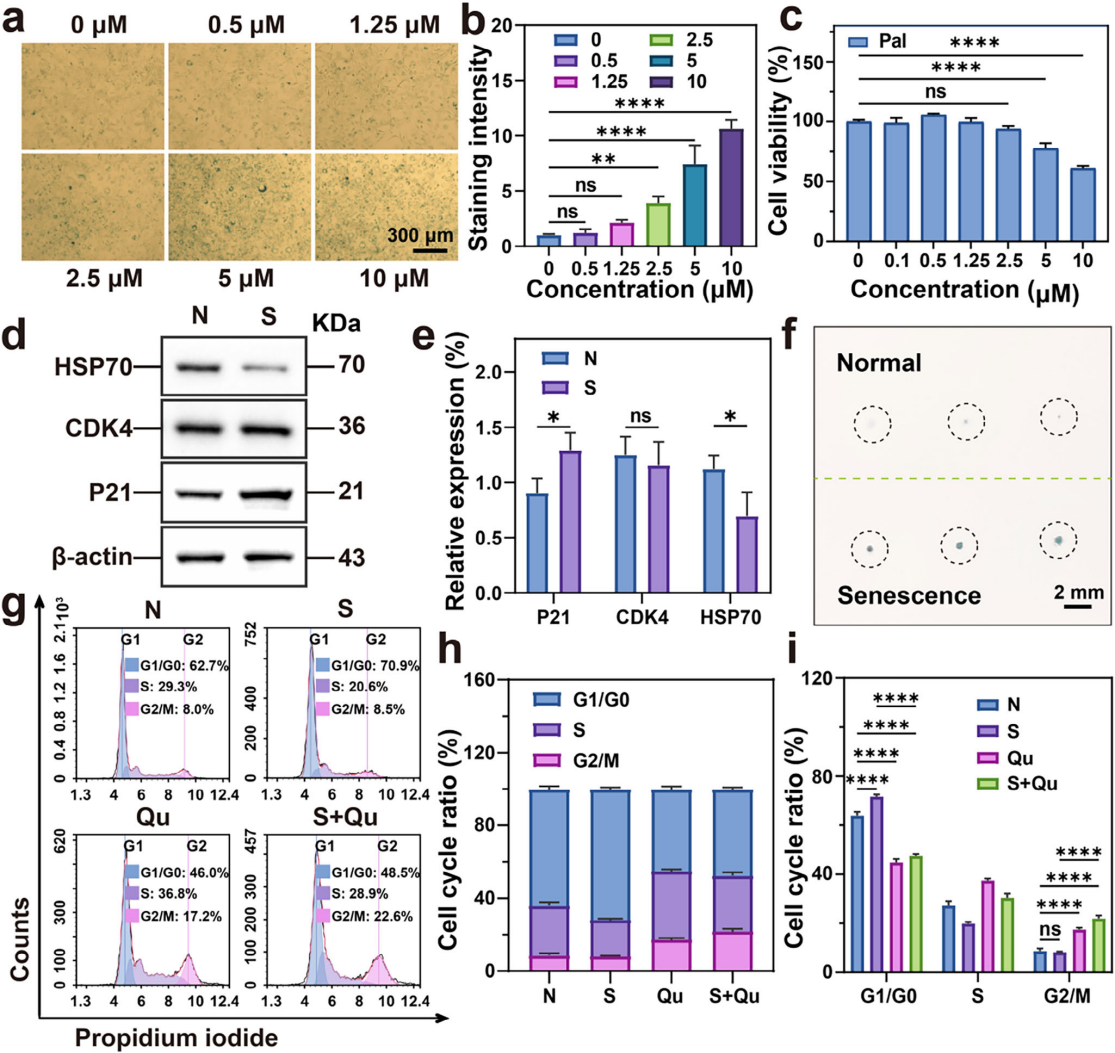
In vitro induced senescence validation. a) Representative images of SA‐β‐gal staining were measured after 3 days of treatment (Green indicates positive staining). b) Quantification of SA‐β‐gal staining (Mean ± S.D., n = 3, two‐way ANOVA, ɑ = 0.05, ****p < 0.00001, **p < 0.001). c) Cell toxicity validation of pal (Mean ± S.D., *n* = 3, one‐way ANOVA test, ɑ = 0.05, ****p < 0.00001). d) Representative western blot results in cells subjected to senescent treatment. e) Western blot analysis (Mean ± S.D., n = 3, two‐way ANOVA, ɑ = 0.05, *p < 0.05). f) Tumor spheroids SA‐β‐gal staining after inducing senescence. g) Flow cytometry analysis of cell cycle. h) Quantitative results of flow cytometry analysis. i) Statistical results of flow cytometry analysis(Mean ± S.D., n = 3, two‐way ANOVA, ɑ = 0.05, ****p < 0.00001).

We first verified the effects of in vitro induced‐senescence combined with senolytics on cell cycle arrest and the clearance of senescent cells. A normal group (N) and a senescent group (S) were set up. After treating treatment of 4T1 cells with different concentrations of palbociclib (pal) for 3 days, followed by quantitative SA‐β‐galactosidase (SA‐β‐gal) staining (**Figure** **1**a,b), revealed effective senescence induction at 2.5 µM with minimal cytotoxicity (Figure 1c), establishing this concentration for subsequent experiments. Mechanistic studies (Figure 1d,e) revealed a significant increase in P21 expression, while CDK4 levels remained unchanged, likely due to negative feedback regulation. Moreover, HSP70 expression in senescent cells decreased remarkably, in line with our strategy of downregulating HSP expression via senescence to enhance mild PTT. Qu exhibited negligible toxicity to normal cells (Figure S1, Supporting Information) but effectively eliminated senescent cells at 100 µg mL^−1^. The senescence‐inducing effects of pal were further confirmed using tumor spheroid models (Figure 1f). Cell cycle analysis by flow cytometry (Figure 1g–i) revealed that after normalization (G0/G1 + S + G2/M = 100%), while N group cells exhibited natural senescence during proliferation, pal treatment significantly induced G0/G1 phase (71.8% ± 1.0%) arrest in 4T1 cells compared to N group (64.0% ± 1.3%). Notably, Qu monotherapy in normal cells cleared naturally senescent cells similar to S+Qu, demonstrating a substantial increase in S phase (37.5 ± 0.7% in Qu group versus 30.5% ± 1.5% in S+Qu group) and G2/M phase cells (22.1 ± 1.4% in S+Qu group versus 8.3% ± 0.1% in S group). This profound cell cycle shift suggests that senescent cell elimination facilitates cell cycle re‐entry and proliferation competence in residual populations. These data confirm that pal effectively induces senescence and that Qu exhibits senolytic activity, thereby establishing a foundation for further cellular studies.

### 2.2 In Vivo Induced Senescence Validation

**Figure 2:**
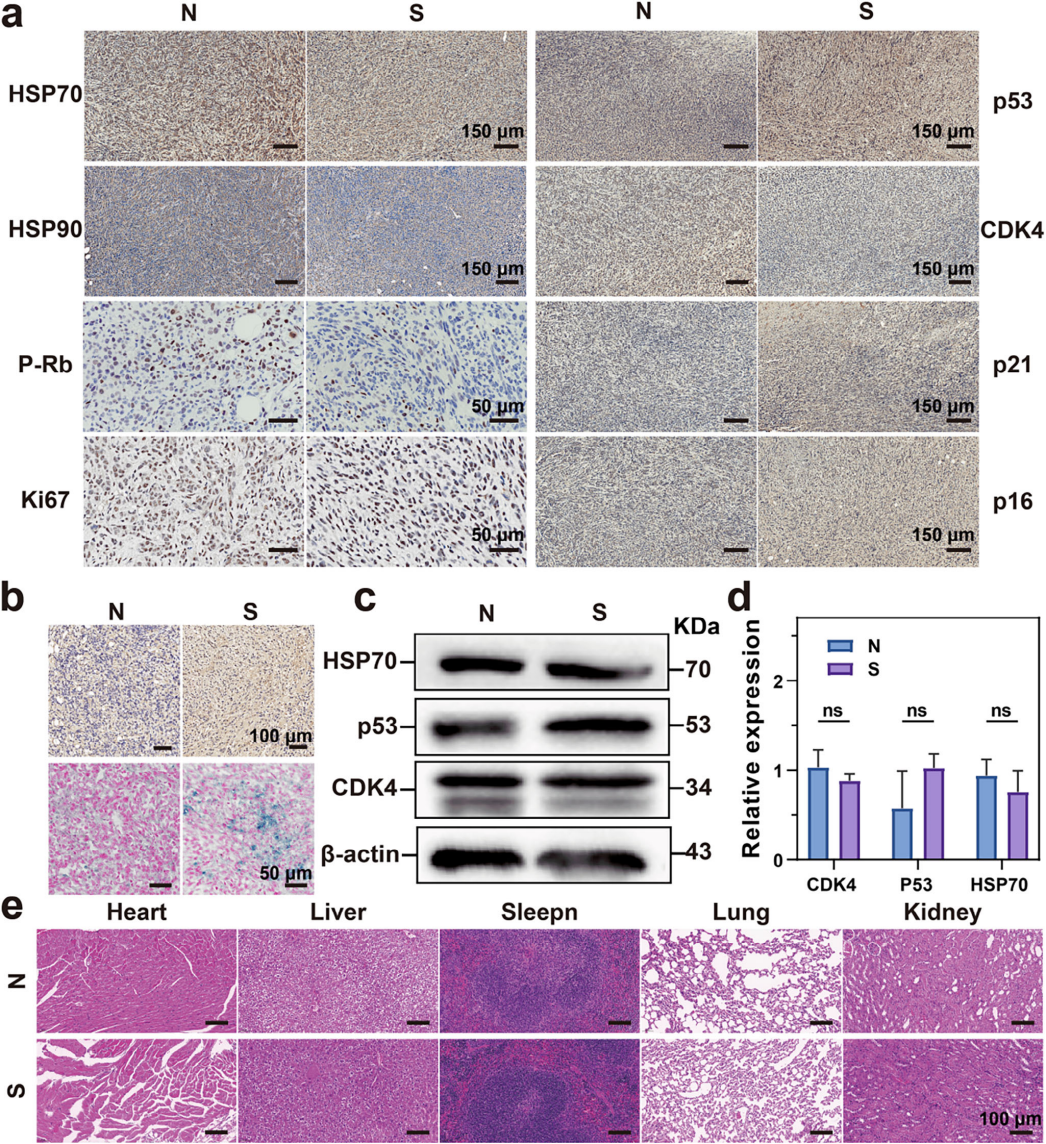
In vivo induced senescence validation. a) Immunohistochemical photographs of senescence related pathways, proliferation related components, as well as HSP expression. b) SA‐β‐gal immunohistochemistry (up) and staining results (down). c) Representative western blot results of CDK4, P53, HSP70 proteins in animal senescence tumors induced by pal gavage. d) Quantitative analysis results of western blot (Mean ± S.D, n = 3, two‐way ANOVA, ɑ = 0.05, ****p < 0.00001). e) H&E staining of heart, liver, spleen, lung and kidney in senescence inducing mice.

To optimize and evaluate the effect of in vivo senescence induction, we gavaged tumor‐bearing mice with a pal suspension at a concentration of 100 mg kg^−1^. After 7 days, we examined the success and safety of establishing a senescent mouse model. First, we conducted immunohistochemical qualitative analysis on the senescence, proliferation, and apoptosis pathways of tumor tissues as well as HSP (**Figure** **2**a). Subsequently, quantitative SA‐β‐gal immunohistochemistry and staining were performed on the tissues (Figure 2b). The results showed that cell senescence could be effectively induced at a concentration of 100 mg kg^−1^. Therefore, this concentration was selected for subsequent experiments. Mechanistic investigations (Figure 2c,d) revealed that senescent tumor tissues exhibited more obvious upregulated p53 expression than modest reductions in CDK4 and HSP70 levels. These molecular alterations are consistent with our therapeutic strategy of leveraging cellular senescence to downregulate HSP expression, thereby potentiating the efficacy of mild PTT. Finally, hematoxylin and eosin (H&E) staining of the heart, liver, spleen, lung, and kidney of the model mice confirmed that this modeling method had good biocompatibility (Figure 2e). These data verified the efficacy of pal in inducing in vivo senescence in tumor‐bearing mice, laying a foundation for further in vivo studies.

### 2.3 Preparation and Characterization of IQ NPs

**Figure 3:**
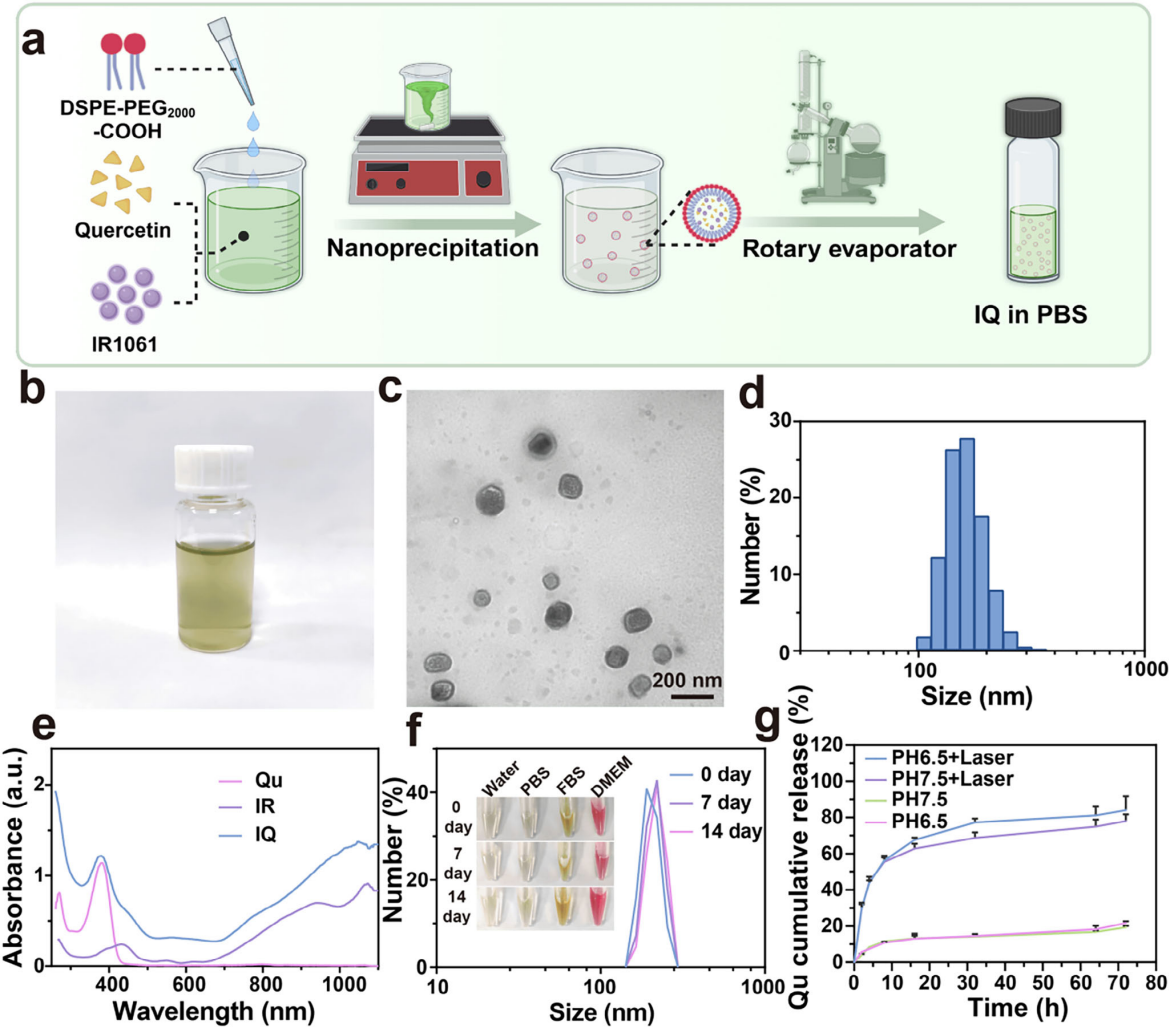
Characterization of IQ NPs. a) Schematic diagram of the preparation process of IQ by the nano co‐precipitation method. b) Images of IQ NPs (50 µg ml^−1^) in PBS. c) Transmission electron microscopy (TEM) of IQ NPs. d) Size distribution for IQ NPs. e) UV–vis‐NIR spectra of Qu and IR 1061 in acetonitrile and IQ NPs in PBS. f) The particle size of IQ NPs (25 µg ml^−1^) in different dispersion media at 0, 7 and 14 days. g) release curves of IQ NPs (70 µg ml^−1^) dispersed in PBS (pH 6.5 and 7.5) with and without laser irradiation conditions (0.8 W cm^−2^, 4 min).

We synthesized IQ NPs via the nano co‐precipitation method. The NIR‐II photosensitizers IR1061 (IR) and Qu served as the functional cores of IQ NPs (**Figure** **3**a). Our findings indicated that IQ NPs demonstrated more favorable NIR‐II absorption characteristics in PBS with a pH of 6.5 compared to PBS at pH 7.5 and pH 5.5 (Figure S2, Supporting Information), it is consistent with the slight acidity of the tumor site, allowing IQ NPs to maintain tumor site stability. Consequently, PBS with a pH value of 6.5 was selected as the dispersion medium for IQ NPs. Initially, to identify the optimal ratio of IR to Qu in IQ NPs, we prepared three batches of IQ NPs with IR:Qu ratios of 1:2, 1:1, and 2:1 respectively. The particle size of 2:1 and 1:1 IQ NPs was ≈190.1 ± 40 nm and 122.4 ± 20 nm, while that of 1:2 IQ NPs measured ≈164.2 ± 20 nm (Figure S3, Supporting Information). The zeta potential of pure Qu was ≈ −25 mV, and that of IR was ≈ ‐20 mV. For IQ NPs with different ratios, the zeta potentials were −6 mV, −20 mV, and −22 mV in sequence (Figure S4, Supporting Information). This clearly shows that IQ NPs with ratios of 1:2 and 2:1 possess higher zeta potentials, suggesting better stability. Subsequently, we measured the drug loading capacity and encapsulation efficiency of IQ NPs with varying ratios. The drug loading rates of IR in IQ NPs with different ratios were 20.44%, 23.04%, and 37.98% respectively, while those of Qu were 19.58%, 36.59%, and 20.45% respectively (Figure S5, Supporting Information). Regarding the encapsulation efficiency, that of IR was 87.12%, 83.86%, and 88.98% respectively, and that of Qu was 64.25%, 76.43%, and 46.36% respectively (Figure S6, Supporting Information). Based on these results, compared with 1:1 IQ NPs, 1:2 IQ NPs presented better drug loading performance for IR. In contrast to 2:1 IQ NPs, 1:2 IQ NPs demonstrated superior drug loading capabilities for Qu. Moreover, a lower dosage of IR can effectively mitigate the toxicity associated with high‐dose organic dye IR, thereby ensuring enhanced biological safety of the NPs. Additionally, the encapsulation of IR in 1:2 IQ NPs was comparable to that in 1:1 and 2:1 IQ NPs, while the encapsulation of Qu in 1:2 IQ NPs was more efficient than in the other two ratios of IQ NPs. Therefore, we determined that IQ NPs with an IR:Qu ratio of 1:2 represent the optimal core ratio. The prepared 1:2 NPs nanoparticle solution manifested as a clear and transparent yellow‐green liquid (Figure 3b). IQ NPs were uniformly dispersed in PBS, forming spherical particles with a diameter of ≈164.2 ± 30 nm (Figure 3c,d). From the UV–vis‐NIR spectra, the maximum absorption peak of Qu was detected at 382 nm, and that of IR was found at 1073 nm, validating the successful co‐loading of IR and Qu (Figure 3e). Subsequently, IQ NPs were diluted to a concentration of 25 µg ml^−1^ in water, PBS, fetal bovine serum (FBS), and Dulbecco's Modified Eagle Medium (DMEM). The particle size of IQ NPs increased from 190.1 ± 30 nm on day 0 to 220 ± 30 nm on day 14 (Figure 3f), and no significant precipitation occurred. This indicates that the NPs exhibit excellent stability. Finally, we systematically evaluated the cumulative release profile of Qu from IQ NPs under physiologically relevant conditions to assess their TME‐responsive drug delivery characteristics. IQ NP suspensions (70 µg ml^−1^) were aliquoted and incubated in PBS at pH 6.5 and 7.5 to simulate the physiological pH and acidic TME conditions, with or without laser irradiation. As demonstrated in Figure 3g, Laser activation triggered an initial burst release phase (> 53.18% within 8 h), as revealed by the release kinetics under both pH conditions and pH‐dependent sustained release profiles, with significantly enhanced cumulative release at acidic pH (77.49 ± 2.15% at pH 6.5 versus 75.14 ± 1.89% at pH 7.4 after 72 h) and accelerated drug release kinetics in acidic conditions, which may be attributed to competitive binding interactions between protonated PEG termini and Qu molecules. These results collectively demonstrate the laser‐responsive release properties of IQ NPs. Importantly, our comprehensive characterization confirms that IQ NPs possess optimal physicochemical properties, including monodisperse size distribution, exceptional colloidal stability, and favorable controlled‐release kinetics, thereby establishing a robust platform for subsequent in vivo therapeutic evaluation.

### 2.4 Photothermal Performance of IQ NPs

**Figure 4:**
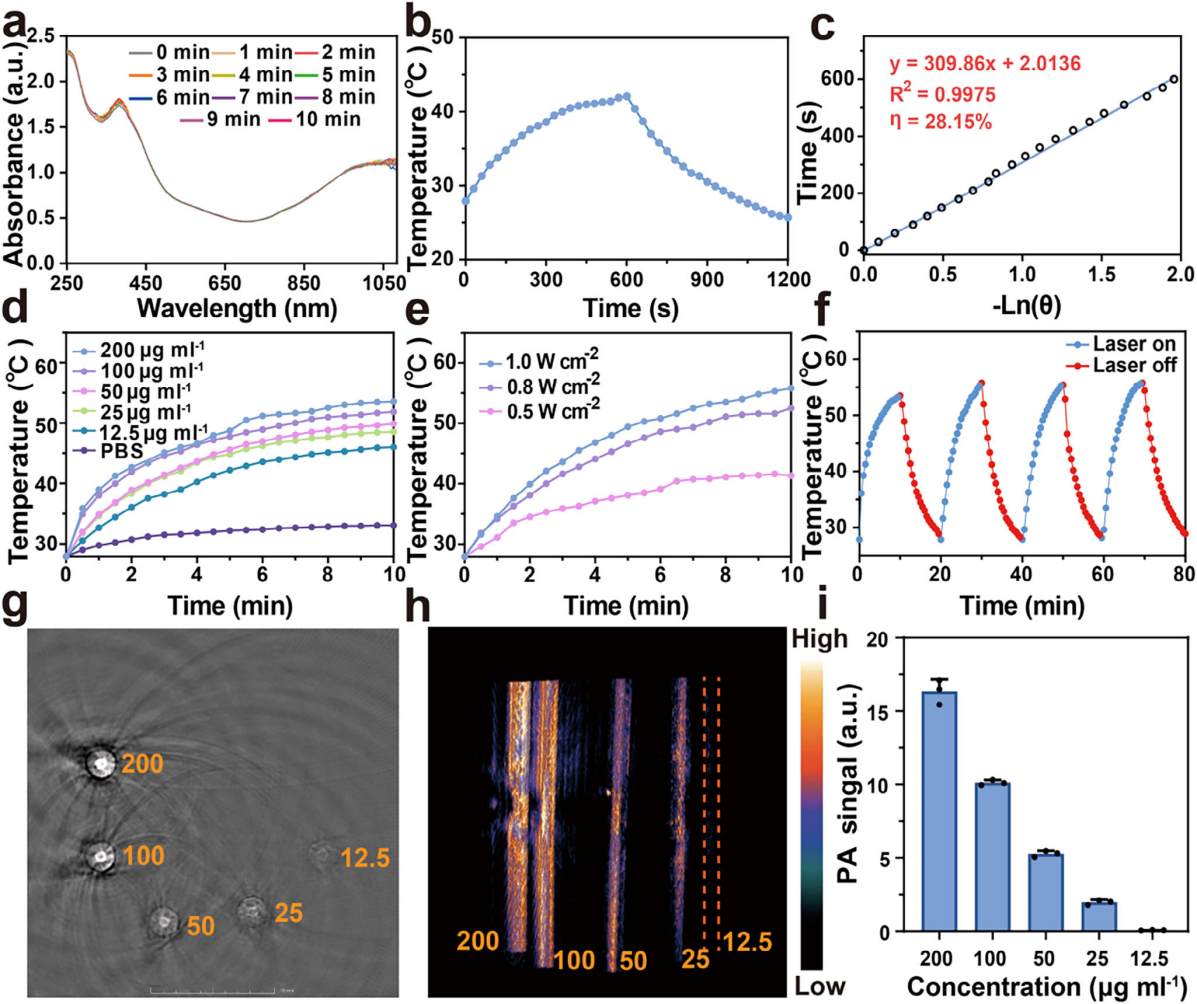
Validation of the optical properties of IQ NPs in vitro. a) The photostability of nanomaterials after 10 min of irradiation with 1060 nm laser (1.0 W cm^−1^). b) Calculation of the PCE of IQ NPs. c) Photothermal fitting curve of IQ NPs under 1060 nm laser excitation. d) Temperature rise diagram of IQ NPs with different concentrations under 1060 nm, 1.0 W cm^−2^ laser irradiation for 10 min. e) Temperature rise diagram of IQ NPs (200 µg ml^−1^) under irradiation of 1060 nm lasers with different power density. f) Temperature change of IQ NPs after 4 cycles (on/off) irradiation by 1060 nm laser (1.0 W cm^−2^). g) PAI of IQ NPs with series of concentrations ranged from 12.5 to 200 µg mL^−1^. h) Vertical view of PAI with series of concentrations ranged from 0 to 200 µg mL^−1^. i) Quantitative analysis of PA signal.

To evaluate the photothermal ability of IQ NPs, the temperature rise under different concentrations and laser intensities with 1060 nm laser excitation was measured using an infrared thermal imager. IQ NPs (100 µg mL^−1^) exhibited stable NIR absorption and good photostability under 1060 nm laser irradiation for 10 min (**Figure** **4**a). Meanwhile, the photothermal conversion efficiency (PCE)^[^ ^34^, ^35^ ^]^ of IQ NPs was evaluated with PBS as a blank control (Figure 4b,c). It was calculated that the PCE of IQ NPs was 28.15%, laying the foundation for subsequent studies on anti‐tumor abilities at the cellular and animal levels. The thermal properties of IQ NPs showed a certain concentration dependence. Under a low concentration of 12.5 µg mL^−1^, the temperature of IQ NPs increased to 46.5 °C (Figure 4d). When the concentration reached 200 µg mL^−1^, the temperature could rise to 54.3 °C. Then, the impact of lasers with different powers on the temperature rise of IQ NPs was verified. When the laser power was 1.0 W cm^−^^2^, the temperature rose to 55.8 °C; when the laser power dropped to 0.5 W cm^−^^2^, its temperature increased to 41.3 °C (Figure 4e). To further evaluate the photothermal performance of IQ NPs, four photothermal cycling experiments were conducted on IQ NPs using a 1060 nm laser with a power of 0.8 W cm^−^^2^ for 10 min each time. Then the laser was turned off and the solution was cooled for 10 min. In these four photothermal cycling experiments, IQ NPs could steadily increase the temperature to 53.3 °C or above, which proved that IQ NPs had good photothermal stability (Figure 4f). Finally, the imaging ability of IQ NPs was evaluated. When different concentrations of IQ NPs were excited by a 1060 nm laser, the intensity of PAI (photoacoustic imaging) were proportional to the concentration, indicating that IQ NPs had the characteristics of comprehensive diagnosis and treatment (Figure 4g–i).

### 2.5 Cellular Uptake and Apoptosis‐Inducing Activity In Vitro

**Figure 5:**
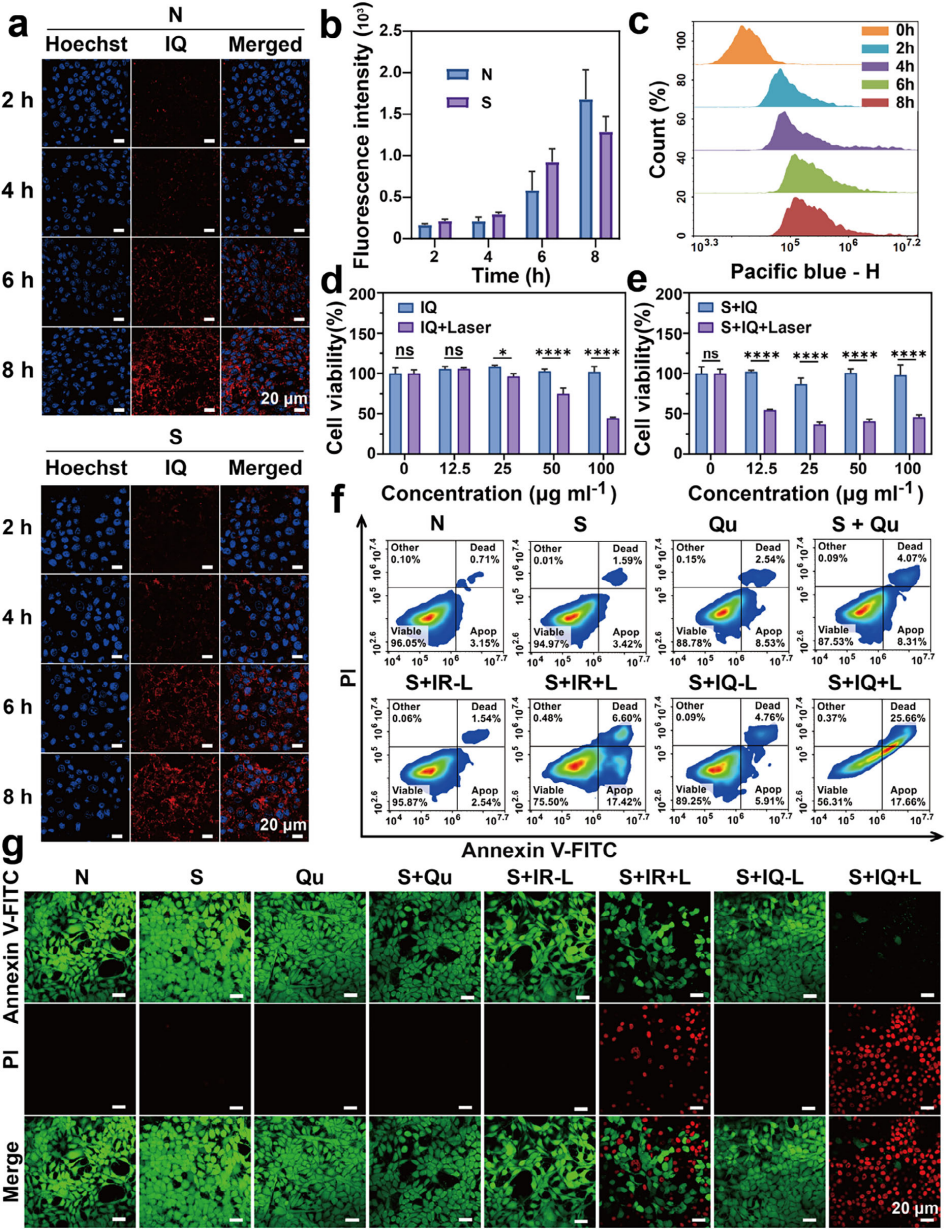
In vitro activity. a) Test the uptake ability of IQ NPs within 8 h. b) Quantitative uptake graph (Mean ± S.D, n = 3, two‐way ANOVA, ɑ = 0.05, *p < 0.05, ****p < 0.00001). c) Quantitative analysis of the uptake capacity by flow cytometry. d) Test the effect of different concentrations of IQ NPs on the survival rate with or without laser irradiation (1060 nm, 1.0 W cm^−2^). e) Test the effect of different concentrations of IR and IQ NPs on the survival rate with or without laser irradiation (1060 nm, 1.0 W cm^−2^) (Mean ± S.D., *n* = 3, two‐way ANOVA test, ɑ = 0.05, *p < 0.05, ****p < 0.00001). f) Representative flow cytometry images showed the level of apoptosis after various treatments (using Annexin V‐FITC/PI apoptosis assay kit). g) CLSM images of 4T1 cells stained by Calcein‐AM (green, live) and PI (red, dead), after different treatments.

Next, we verified the uptake and killing effects of IQ NPs and its respective components in vitro. To comprehensively evaluate the cellular uptake dynamics and penetration capability of IQ NPs, we first investigated their internalization kinetics in 2D 4T1 cell cultures. Confocal laser scanning microscopy (CLSM) and flow cytometry analyses revealed that both N group and S group 4T1 cells showed initial IQ NP (labeled with cy5.5^[^ ^36^, ^37^ ^]^) accumulation within 2–4 h post‐administration, with slightly enhanced uptake observed in senescent cells (**Figure** **5**a–c). The uptake reached maximum in non‐senescent cells within 4–8 h. To better model solid tumor characteristics, we established 3D multicellular tumor spheroids and evaluated IQ NP penetration.^[^ ^38^ ^]^ After incubating non‐senescent and senescent tumor spheroids with Cy5.5‐labeled IQ NPs for 8 h, CLSM z‐stack imaging at 20 µm intervals demonstrated that IQ NPs exhibited good penetration abilities in both spheroid types, with significant fluorescence detected even in core regions (0 µm depth) (Figure S7, Supporting Information). The IQ NPs accumulated throughout the tumor spheroids at 8 h without showing reduced penetration capacity due to cellular metabolic changes. These results demonstrate that IQ NPs can successfully penetrate and accumulate within both non‐senescent and senescent tumor spheroids under NIR‐II laser irradiation conditions, while maintaining consistent uptake kinetics in 2D and 3D models. Therefore, in subsequent cell experiments, 8 h was selected as the administration time. Subsequently, the CCK‐8 assay was used to evaluate the cytotoxicity of IR NPs (Figure S8, Supporting Information) and IQ NPs under senescent conditions (Figure 5d,e). To investigate the correlation between cellular senescence and thermoresistance, we first evaluated the cytotoxicity of IR+L in both non‐senescent and senescent conditions. In non‐senescent cells, treatment with 100 µg mL^−1^ IR resulted in cell viability below 48.3%. In contrast, senescent cells showed significantly reduced thermoresistance, with viability dropping below 66.2% at just 50 µg mL^−1^ IR compared to non‐senescent cells which maintained >76.0% viability at this concentration. These results clearly demonstrate that senescent cells exhibit markedly diminished heat resistance. Building upon these findings, we proceeded to systematically assess the cytotoxic properties of IQ NPs. When the cells were not senescent and without laser irradiation, even at a high concentration of 25 µg mL^−1^, the survival rates of cells in both groups exceeded 80%. In contrast, under senescent conditions, the killing effect of IQ NPs on cells was more significant. When the drug concentration reaches 25 µg mL^−1^, the cell survival rate is less than 50% of that at 12.5 µg mL^−1^, specifically only 31%. Therefore, 25 µg mL^−1^ was set as the cell administration concentration of IQ NPs. To further verify the above results, cell death and apoptosis assays were also carried out under mild PTT. Flow cytometry was used to detect the combined effect of S and Qu, as well as the ability of IQ NPs ± Laser (L) to induce apoptosis (Figure 5f). S and Qu exhibited low toxicity, with apoptosis rates of 4.52% and 9.81% respectively, and the apoptosis rate of the S + Qu group increased to 11.64%. Compared with the N group, S + IR induced partial cell apoptosis through the photothermal effect after laser irradiation. As a HSP inhibitor, Qu effectively eliminate the heat‐resistance effect of tumor cells and improve the photothermal killing effect. After cell senescence, Qu was used to clear senescent cells, leading to an increase in the late‐stage apoptosis rate. Combining with the inhibitory effect of Qu on HSP, the apoptosis rate of the S + IQ group even reached 33.7% after laser irradiation. Finally, through the cell viability and death assay (Figure 5g), it was observed that without laser irradiation, each group of cells could survive well. However, a small amount of red fluorescence appeared in S + IR ± L, indicating that mild PTT after cell senescence could not produce a significant killing effect. In the S + IQ group, obvious red fluorescence appeared in the field of view after laser irradiation, indicating that a large number of cells had died. Based on the above results, IQ NPs utilized the photothermal effect of IR 1061, combined with Qu to clear senescent cells and weaken the heat‐resistance of tumor cells, thus exerting a stronger apoptotic effect at the cellular level, verifying the excellent tumor‐killing ability of S + IQ +L.

### 2.6 In Vitro Inhibition of Migration and Proliferation and Mechanism Exploration

**Figure 6:**
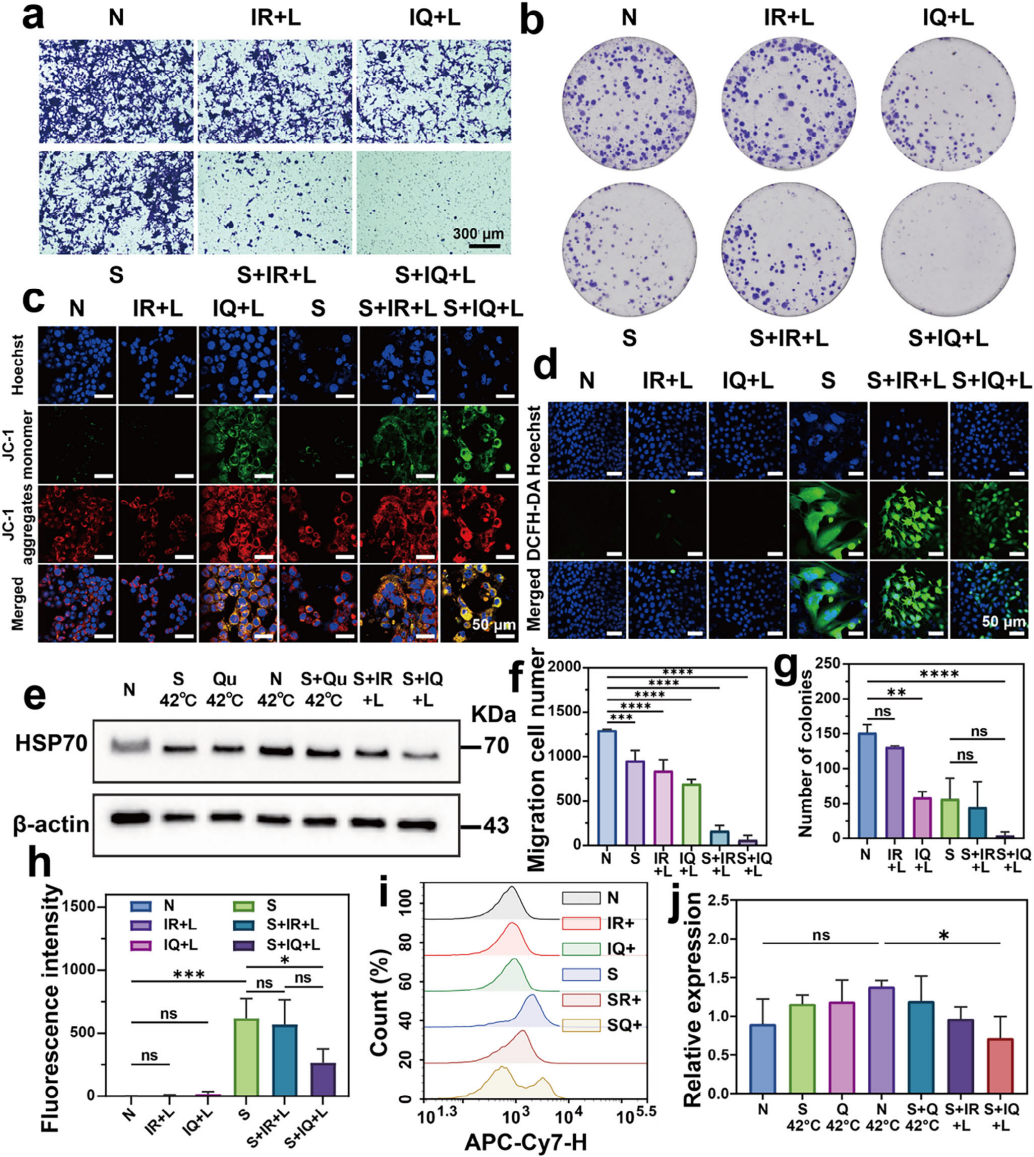
Exploration of the ability and mechanism of in vitro inhibition of migration and proliferation. a) After 24 h, transwell assay was performed with or without laser irradiation of IQ NPs (1060 nm, 1.0 W cm^−2^, 5 min). b) Clone formation assay with or without laser irradiation (1060 nm, 1.0 W cm^−2^, 5 min). c) CLSM images of mitochondrial membrane potential changes in 4T1 cells from different treatment groups (blue fluorescence: Hoechst 33 342, red fluorescence: JC‐1 aggregates, green fluorescence represents JC‐1 monomers, scale bar = 50 µm). d) CLSM images of ROS generation in different treatment groups with and without laser irradiation(1060 nm, 1.0 W cm^−2^, 5 min, scale bar = 50 µm). e) Western blot of HSP70 in 4T1 cells after receiving different treatment conditions. f) Quantitative data of transwell (Mean ± S.D., *n* = 3, one‐way ANOVA, ɑ = 0.05, ***p < 0.001, ****p < 0.00001). g) Quantitative data of colony formation assays. j) Quantitative analysis results of western blot.

To investigate the anti‐tumor potential of S, Qu and IQ NPs, we systematically evaluated their effects on tumor cell proliferation and migration through Transwell chamber assays and colony formation tests. After 3 days of induced senescence, cells were treated with various formulations. The results demonstrated that both IR+L and IQ+L treatments significantly inhibited tumor cell migration (**Figure** **6**a,f), with the S+IQ+L group showing the most potent inhibitory effect. Similarly, colony formation assays revealed markedly reduced proliferation following laser irradiation (Figure 6b,g), where senescent cells formed smaller colonies and the S+IR+L/S+IQ+L treatments exhibited superior anti‐proliferative activity. These findings suggest that the S+IQ+L combination could effectively suppress tumor metastasis and recurrence during mild PTT. Mechanistic investigations showed that under laser irradiation at ≤ 45 °C, IQ+L treatment induced partial mitochondrial membrane potential depolarization compared to IR+L (Figure 6c), indicating HSP regulation mediated by Qu. The enhanced green fluorescence in the S+IQ+L group suggested synergistic HSP modulation through the combined action of senescence and Qu, promoting mitochondrial damage and apoptosis under mild hyperthermia conditions. ROS generation assays (Figure 6d,h,i) demonstrated that while IR itself lacked significant ROS‐producing capacity, senescent cells (S and S+IR+L groups) showed substantial ROS accumulation, which was effectively scavenged by Qu in the S+IQ+L group. Western blot analysis (Figure 6e,j) confirmed that heat treatment at 42 °C for 20 min upregulated HSP70 expression compared to controls, while the S 42 °C and Qu 42 °C groups showed significant HSP70 downregulation. Notably, the S+IQ+L treatment exhibited the lowest thermotolerance and HSP70 expression levels, likely resulting from combined cellular damage induced by the synergistic therapy. These comprehensive results demonstrate that the S+IQ+L combination effectively inhibits tumor cell migration and proliferation through coordinated mechanisms involving mitochondrial dysfunction induction, ROS regulation, and HSP70 suppression. The dual action of Qu as both an HSP inhibitor and senolytic agent, combined with senescence induction and mild PTT, establishes a potent multimodal anti‐tumor strategy with strong potential for preventing metastasis and recurrence. These findings provide a solid mechanistic foundation for further in vivo investigations.

### 2.7 In Vivo Therapeutic Evaluation in a TNBC Mouse Model

**Figure 7:**
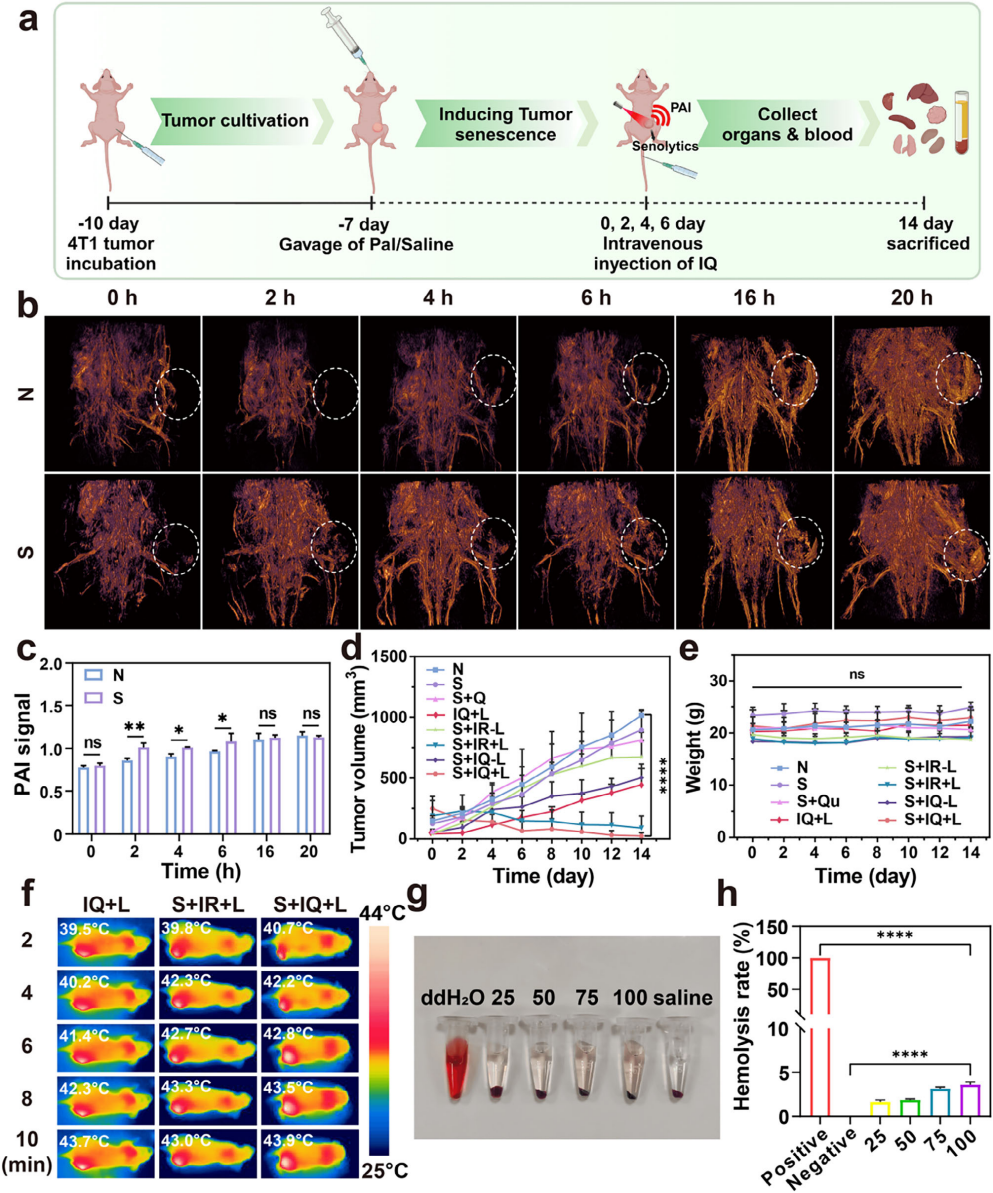
The inhibitory effect of laser irradiation on IQ NPs in senescence subcutaneous tumor‐bearing mice. a) The treatment plan for subcutaneous tumor bearing mice. b) Time dependence of PAI at tumor site after intravenous injection of IQ NPs in normal and senescence tumor‐bearing mice. c) Quantification of PA signal. d) Changes in tumor volume during mouse treatment. (Mean ± S.D., *n* = 5, two‐way ANOVA test, ɑ = 0.05, ****p < 0.00001). e) Weight change chart of mice during treatment. f) Changes in thermal imaging of mice treated with IQ+L S+IR+L and S+IQ+L over time under 1060 nm laser irradiation (1.0 W cm^−2^). g) Images of hemolysis experiments with different concentrations of IQ NPs. h) Quantification of hemolysis experiment (Mean ± S.D., *n* = 3, one‐way ANOVA test, ɑ = 0.05, ****p < 0.00001).

Based on the pre‐experiment on inducing tumor senescence in vivo, we established a mouse model bearing senescent tumors. To further verify the tumor‐targeted accumulation and oncolytic effect of IQ NPs, we injected IQ NPs into mice via the tail vein and monitored the accumulation of IQ NPs signals at tumor sites as well as the changes in tumor size and body weight of the mice. Finally, the mice were euthanized (**Figure** **7**a). First, we verified the targeting and imaging capabilities of IQ NPs in mice of N group and S group. The PAI and quantification of TNBC model mice after tail vein administration of IQ NPs (with IR1061 at 10 µg) were obtained (Figure 7b,c). The PA signals indicated that during the early stage of tail vein injection, the S group mice had better uptake of IQ NPs. This might be due to the decreased defensive capabilities of senescent tumor cells, which made it easier for IQ NPs to penetrate. Meanwhile, during the later stage of tail vein injection, the accumulation in the S group and the N group was similar, and both groups reached the maximum uptake of IQ NPs. Therefore, IQ NPs could accumulate well in tumors within 20 h and had good PAI effects. Second, after inducing senescence, N (PBS), Qu, IR NPs (with IR1061 at 10 µg), and IQ NPs (with IR1061 at 10 µg) were injected via the tail vein, and then the tumors were irradiated with a 1060 nm laser (1.0 W cm^−^^2^, 10 min). The 14‐day treatment regimen revealed progressive tumor growth inhibition across all experimental groups. The N group showed rapid tumor progression with a mean final volume of 1016.0 mm^3^, while the S group exhibited moderate growth suppression with tumors reaching 895.6 mm^3^. In comparison, the S+Qu group demonstrated further reduction to 812.7 mm^3^, with significantly attenuated growth kinetics indicating combined effects of S and Qu. The IQ+L group, representing non‐senescent tumors receiving mild PTT, achieved a mean final volume of 444.9 mm^3^, confirming Qu's ability to enhance mild PTT efficacy. Most remarkably, the S+IR+L regimen resulted in substantial tumor ablation with a final volume of 154.6 mm^3^, while the comprehensive S+IQ+L approach combining all therapeutic modalities achieved near‐complete tumor eradication at just 23.7 mm^3^ compared to the N group in Figure 7d. This demonstrated the excellent anti‐tumor effect achieved by inducing tumor senescence, reducing thermal tolerance, and supplementing with senescence scavengers and HSP inhibitors to strengthen mild PTT, with no significant body weight changes observed in treated mice (Figure 7e). These findings systematically demonstrate the therapeutic advantage of sequentially combining cellular senescence with quercetin‐mediated pathways and mild photothermal ablation. By monitoring the changes in tumor temperature, the efficiency of mild PTT after laser irradiation was studied. After laser irradiation, the body temperature at the tumor sites of the mice was within the temperature range of mild PTT, indicating the mild PTT efficiency of IQ NPs in vivo (Figure 7f). Finally, the hemolysis‐related biological safety of IQ NPs in vivo was verified. As the concentration of IQ NPs increased, the hemolysis rate increased from 1.65% to 3.62%. Considering the possible dissolution of nanoparticles during the experiment, the actual hemolysis rate should be lower (Figure 7g,h). The above experiments demonstrated the good, targeted accumulation and PAI capabilities of IQ NPs. In the in vivo treatment experiments, the S + IQ + L group exhibited the best anti‐tumor effect and stable biochemical safety.

### 2.8 Biological Safety Assessment of In Vivo Therapy

**Figure 8:**
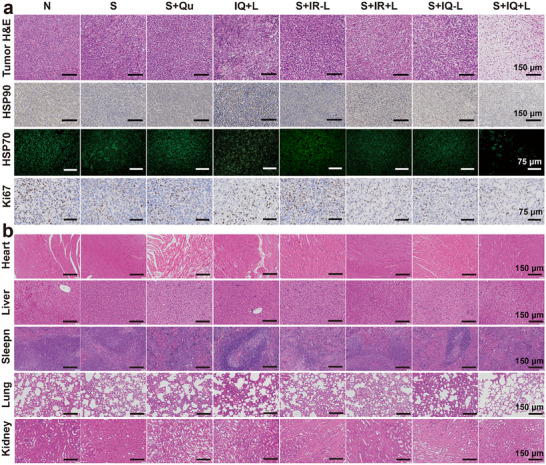
Antitumor ability and biosafety. a) H&E, HSP70, HSP90, Ki67 staining images of tumor from different treated mice. b) H&E staining images of main organs from different treated mice.

Following completion of the in vivo treatment protocol, we conducted systematic biosafety evaluations through comprehensive analysis of collected organs, blood samples, and tumor tissues from all experimental groups. Histopathological assessment of tumor sections using H&E staining demonstrated marked ablation features specifically in the S+IQ+L group, corresponding to its exceptional therapeutic performance, while other treatment groups displayed no significant tumor morphological changes. Immunohistochemical analysis revealed stable expression levels of HSP70 and HSP90 in both the N group and S+Qu group, with other treatment groups showing gradually decreasing expression patterns that precisely matched our in vitro mechanistic results. These therapeutic effects were further confirmed by proliferation marker Ki67 staining (**Figure** **8**a). All treatment approaches exhibited excellent biocompatibility. Hematological and biochemical parameters (ALT, AST, BUN, CREA) showed no significant alterations, with all values within normal ranges. The Qu group displayed slightly increased ALT and AST levels that remained within normal limits, possibly due to tail vein administration of quercetin (Figure S9, Supporting Information). Thorough histological examination of major organs revealed no pathological changes associated with treatment in any experimental group (Figure S9, Supporting Information). These findings collectively demonstrate the outstanding biosafety characteristics of the IQ nanoparticle‐based therapeutic platform.

## 3 Conclusion

In conclusion, our study has successfully integrated senescence‐inducing therapy with NIR‐II mild PTT and senolytic agents for TNBC treatment, overcoming the limitations of conventional therapies through synergistic effects. We developed a novel nanodrug system, IQ NPs, for efficient TNBC phototherapy, establishing a new treatment paradigm. The efficacy and safety of pal in inducing TNBC senescence were comprehensively validated through both in vitro and in vivo experiments. The engineered IQ NPs demonstrated high drug‐loading capacity, excellent encapsulation efficiency, and remarkable photostability, enabling effective multimodal combination therapy. In vitro studies confirmed that IQ NPs under NIR‐II laser irradiation significantly inhibited tumor cell proliferation and migration while promoting apoptosis. Qu further enhanced therapeutic outcomes by reducing HSP expression to potentiate mild PTT effects while simultaneously clearing senescent cells. In vivo evaluations demonstrated effective accumulation and retention of IQ NPs at tumor sites under NIR‐II PAI guidance. Upon 1060 nm laser irradiation, IQ NPs elevated tumor temperature to ≈44 °C, achieving effective mild thermal ablation. The combined S+IQ+L therapy demonstrated superior tumor growth inhibition without inducing significant adverse effects, confirming both therapeutic efficacy and biosafety. Although preclinical results demonstrate promising biocompatibility, clinical translation faces challenges, including the potential impact of TNBC heterogeneity on treatment generalizability, possible development of drug resistance, and the complexity of implementing multimodal therapies. Future research should prioritize the optimization of personalized treatment regimens, exploration of immunotherapy combinations, and simplification of technical approaches to facilitate clinical adoption. This innovative therapeutic strategy capitalizes on the complementary advantages of sequential therapies to effectively eliminate tumor cells while minimizing damage to normal tissues, representing a groundbreaking approach for TNBC treatment.

## 4 Experimental Section

### Materials and Instrumentation

The chemical reagents used in the above experiments were purchased from Adamas Co., Ltd (Shanghai, China). IR1061 was purchased from Macklin Co., Ltd (Shanghai, China). DSPE‐PEG_2000_‐COOH was purchased from Nanosoft Polymers (USA). Cy5.5 was purchased from Yuanye Biotechnology Co., Ltd (Shanghai, China). Mouse 4T1 cells (TCM32) were purchased from QuiCell Biotechnology Co., Ltd (Shanghai). CCK‐8, 1% penicillin‐streptomycin and High glycemic DMEM were purchased from Yeasen Science & Technology Co., Ltd (Shanghai, China). Calcein‐AM/PI Cell Viability/Cytotoxicity Assay Kit, Annexin VFITC Apoptosis Detection Kit, Hoechst 33 342 (1000×) were purchased from Beyotime Biotechnology Co., Ltd. Annexin V‐FITC/PI was purchased from SONY. The primary antibodies for HSP70, CDK4, P21, P53, β‐actin and secondary antibodies were purchased from Cell Signaling Technology (USA). All culture media contained 10% fetal bovine serum (FBS, Biological Industries). Unless otherwise mentioned, all solvents used were deionized water. The size and shape of IQ NPs were observed using TEM (HT7700, Hitachi, Japan). The UV–vis‐NIR absorption spectra of the nanoparticles were tested using a spectrophotometer (Thermo Fisher). The size distribution of IQ NPs was evaluated by a Zetasizer nano ZS particle analyzer (Malvern Instruments Co., Ltd.). Cell flow cytometry experiments were analyzed by using Agilent NovoCyte Quanteon analyzer. Fluorescence images were detected using CLSM (Leica DMI8). In vitro and in vivo PAI were collected by TOMOWAVE (Suzhou) medical imaging Co., Ltd. The thermal maps were provided by a thermal imager (FLUKE, TI400).

### Preparation of IQ NPs

IQ NPs was prepared by nano co‐precipitation method using IR1061 (1 mg), Qu (2 mg), and DSPE‐PEG_2000_‐COOH (3 mg). Specifically, IR1061 (1 mg), Qu (2 mg), and DSPE‐PEG_2000_‐COOH (3 mg) were severally dissolved in 2 mL of ACN, and the above liquids were placed in an ultrasonic water bath for 10 min. Then, the three liquids were mixed and stirred overnight at room temperature and a stirring rate of 1000 rpm in a dark environment. Use rotary distillation to remove ACN, then add PBS (PH 6.5) solution to obtain IQ NPs, and store it in a refrigerator at 4 °C for the next experiment. 1: 1 IQ NPs and 2: 1 IQ NPs were also prepared using a similar preparation method.

### Characterization of IQ NPs

Observe the morphology of the prepared IQ NPs through TEM and perform size analysis. Measure the size distribution of IQ NPs and calculate surface potential using Zetasizer nano ZS particle analyzer. Measure the UV‐Vis‐NIR absorption spectrum of IQ NPs using a UV–vis‐NIR spectrophotometer. In vitro stability of IQ NPs in PBS was monitored by Zetasizer Nano ZS particle analyzer on the 0 day, 7th day, and 14th day after the IQ NPs was synthesized.

### Drug Loading Rate and Encapsulation Rate

A certain volume of fixed concentration IQ NPs solution was prepared. Standard curves of IR and Qu were made, and the UV–vis‐NIR absorption value of the supernatant of IQ NPs was measured to calculate the content of IR and Qu in IQ NPs. The encapsulation rate (ER) and drug loading rate (DL) were calculated as:

(1) $$ER\left(\%\right)=\frac{W_{e}}{W_{f}}\times 100\%$$

(2) $$DL\;\left(\%\right)=\frac{W_{e}}{W_{s}}\times 100\%$$

(*W_e_* is the mass of IR and Qu in IQ NPs; *W_f_* is the total amount of drugs; and *W_s_* is the total mass of IQ NPs.)

### Photostability

1 ml of IQ NPs (100 µg mL^−1^) solution was added to a quartz dish and irradiated with 1060 nm laser (1.0 W cm^−2^) for different times (0, 1, 2, 3, 4, 5, 6, 7, 8, 9, and 10 min), and then the changes of UV–vis‐NIR absorption spectra were measured by UV–vis‐NIR spectrophotometer for evaluation of IQ NPs photostability.

### In Vitro Cumulative Release of Qu

Centrifuge the newly prepared IQ NPs at 12 000 rpm for 5 min to remove the supernatant, and then supplement them with PBS (pH 6.5 and pH 7.5) to the original volume. Collect IQ NPs from the release medium (PBS, pH 6.5 and pH 7.5) at time intervals (0, 2, 4, 8, 16, 32, 64, and 72 h) and centrifuge at 12 000 rpm for 5 min with or without laser irradiation to precipitate the nanoparticles. Take aliquots of the supernatant (e.g., 1 mL). Add an equal volume of PBS each time to maintain a constant system solution volume. Carefully transfer the obtained supernatant onto a 96 well plate and measure the UV absorption using a UV–vis NIR enzyme‐linked immunosorbent assay reader at a wavelength of 380 nm. Substitute it into the quercetin concentration absorption standard curve and calculate the cumulative release amount.

### Photothermal Performance and Photothermal Stability of IQ NPs

1) To investigate the photothermal properties of IQ NPs, the solutions with different IQ NPs concentrations (0, 12.5, 25, 50, 100 and 200 µg mL^−1^, 1 ml) were irradiated for 600 s using a 1060 nm laser (1 W cm^−2^), and the temperatures were recorded with a thermal imaging camera every 30 s.

2) The 1060 nm laser was set to different powers (0.5, 0.8 and 1 W cm^−2^) to irradiate the IQ NPs (200 µg mL^−1^, 1 ml) solution for 600 s, and the temperatures were recorded every 30 s to evaluate the effect of different powers on the photothermal properties of NPs.

3) In addition, 12.5 µg ml^−1^ of IQ NPs solution was added into a quartz dish and alternately heated (laser on 600 s) and cooled (laser off 600 s) for four cycles using 1060 nm laser (0.8 W cm^−2^) to study the photothermal stability of IQ NPs. As reported heretofore, the photothermal conversion efficiency was calculated.

The heating and cooling curves of the aqueous nanoparticle solution were plotted. The PCE of IQ NPs was calculated by equation (3):

(3) $$\eta =\frac{hA\left(\Delta T_{max,NPs}-\Delta T_{max,H_{2}O}\right)}{I\left(1-10^{-A_{\lambda }}\right)}$$

where *h* and *A* represent heat transfer coefficient and surface area of vessel, *T_max_* represents temperature change of IQ NPs and H_2_O at maximum steady‐state temperature, *I* represents laser power (1.0 W cm^−2^), *A_λ_* is the maximum absorption wavelength of NPs.

### PAI Performance

In vivo and in vitro PAI experiments were carried out in LOIS‐3D PAI equipment provided by TOMOWAVE (Suzhou) medical imaging Co., Ltd. For in vitro PA intensity test, diluting IQ NPs into solutions of different concentrations (12.5, 25, 50, 100, and 200 µg ml^−1^) with PBS, and then excite the sample under 1060 nm laser., and then the sample was excited under 1060 nm laser. For in vivo PAI, tumor‐bearing nude mice were anesthetized with isoflurane, and IQ NPs (IR 100 µg) were injected into the mice through the tail vein. At different time points, PAI were collected under 1060 nm laser excitation.

### Cell Culture

4T1 cells (mouse breast cancer cells) were incubated in high‐sugar DMEM containing 10% FBS, 1% penicillin, and 1% streptomycin in a 5% CO_2_ incubator at 37 °C. The medium was changed every 2–3 days. When the number of cells exceeded 90%, the cells were digested with 0.25% trypsin EDTA solution. After 2–3 min of digestion, the digestion was terminated with high‐sugar DMEM, centrifuged at 1000 rpm for 3 min, and finally the cells were passaged.

### SA‐β‐gal Activity Assay

To verify the effect of pal on senescence induction, the expression of SA‐β‐gal in senescent cells and tumor spheroids was measured by using SA‐β‐gal staining kit (Beyotime Biotechnology, Shanghai, China). In short, 4T1 cells (5×10^4^ per well) were seeded on a 6‐well plate or multicellular spheroids were cultured in small dishes, incubated for 12 h, and treated with different concentrations and doses of pal (0, 0.5, 1.25, 25 5, and 10 µM) for 3 days. Then wash twice with PBS, fix with 0.5% paraformaldehyde for 15 min, and stain the cells and tumor balls overnight at 37 °C without CO_2_ using SA‐β‐gal staining solution. Finally, observe under APX 100 (Olympus). Quantify senescence cells by measuring the degree of staining of cells and tumor spheroids in each well.

### Flow Cytometry Analysis of Cell Cycle

To verify the effect of senescence on the cell cycle and the clearance effect of Qu on pal induced senescence, we used flow cytometry to analyze the cell cycle. First, divide the cells into N, S, Qu and S + Qu groups before administration. Next, cells were seeded at a density of 1 × 10^5^ per well in a 6‐well plate. The S group treated cells with pal for 3 days and Qu for 8 h. Then gently wash the cells multiple times with PBS, collect the cells and fix them in cold 75% ethanol for at least 4 h or overnight. After multiple gentle washes with PBS for three times, the cells were stained using the Cell Cycle and Apoptosis Analysis Kit (Lablead Biotech CO., LTD, Beijing) and analyzed using an Agilent NovoCyte Quanteon analyzer. The percentages of cells in G0/G1, S, and G2/M phases were normalized to 100% of total viable cell population by proportional scaling, with sub‐G1 debris and apoptotic cells excluded from the cell cycle quantification.

### In Vitro Cellular Uptake

Inoculate 4T1 cells at a density of 1 × 10^5^ cells in a confocal dish and culture for 12 h. Then, the cells were incubated with IQ NPs for 2, 4, 6, and 8 h by changing the culture medium containing IQ NPs (25 µg mL^−1^, containing cy5.5 dye). The cell nucleus was stained with Hoechst 33 342 for 10 min, and then observed using confocal microscopy (LEICA DMI8). Similarly, 4T1 cells were uniformly seeded in a 6‐well plate with 5×10^4^ cells per well and cultured for 12 h. Then replace the nanoparticle culture medium with IQ NPs (containing cy5.5 dye), incubate the cells with IQ NPs for 0, 2, 4, 6, and 8 h, and quantify the dye fluorescence using Flow Cytometer (Agilent).

### Cell Toxicity Experiment

Complete CCK‐8 experiment to detect the cell toxicity of nanomedicine under laser or non‐laser conditions. In short, 4T1 cells were seeded into a 96‐well plate at a rate of 5 × 10^3^ cells per well and cultured for 12 h. Then, cells were treated with different concentrations of S, Qu, IR, IQ, S + IR, S + IQ for 24 h. After washing with PBS and replacing the culture medium with fresh medium, the cells were irradiated with or without 1060 nm laser for 5 min (1.0 W cm^−2^) and cultured for another 24 h. Then, the cell survival rate was detected by CCK‐8 (Yeasen Biotechnology, China) according to the manufacturer's protocol.

### Apoptosis Analysis

4T1 cells were seeded onto a plate at a density of 1 × 10^5^ cells per well and cultured for 24 h. Then, cells were treated with 2.5 µM pal at the same concentration to induce cell senescence for 3 days. After incubation with Qu, IR, and IQ NPs for 8 h, the cells were washed with PBS and irradiated with or without 1060 nm laser for 5 min (1.0 W cm^−2^). Collect these cells and stain them with Annexin V‐FITC/Propidium Iodide (SONY, China). Subsequently, apoptosis efficiency was measured by flow cytometry analysis. For live/dead assay imaging, 4T1 cells treated with nanomedicine were incubated with a Calcein‐AM/PI Live/Dead Viability Cytotoxicity AssayKit (Beyotime Biotechnology, China) and observed using CLSM.

### Transwell Assay

In the study, 24 wells per well were used as transwell units (Corning, 3422) to test the migration ability of tumor cells. After processing, 4 × 10^4^ 4T1 cells were seeded in the upper chamber of serum‐free medium, while the lower chamber was filled with medium containing 10% FBS and cultured for 24 h. Then, pal was co‐cultured with cells for 3 days to induce cell senescence. Then, IR and IQ NPs serum free medium was used to replace the culture medium in the upper chamber. After 8 h of cultivation, laser treatment or no laser treatment was performed. After another 2 h of incubation, cells were washed with PBS. Then fix the cells in the lower layer of the chamber with cell fixative, stain the cells in the lower layer of the chamber with crystal violet dye for 10 min, observe the bottom of the chamber using APX 100 (Olympus), and count and analyze the cells at the bottom of the membrane using Image J.

### Cell Colony Formation Assay

To test the effects of senescence and IQ on cell proliferation and killing, 4T1 cells were seeded into a 6‐well plate with 700 cells per well. After one week of cultivation, add the corresponding drug to each well and perform the same administration treatment as before, with or without laser treatment, and then incubate for 24 h. Finally, fix the cell colonies with 4% paraformaldehyde, stain with 0.5% crystallization solution, and observe under a microscope and analyze by Image J.

### Intracellular ROS Detection

Evaluate the effects of S, Qu, IR, and IQ components on ROS production in cells using DCFH‐DA fluorescent probes. Inoculate 4T1 cells at a density of 1 × 10^5^ into a 4‐chamber glass bottomed culture dish and incubate under standard conditions for 24 h to allow cell attachment. Subsequently, the aging group cells were induced to senescence for 3 days, and then subjected to further incubation for 3 h with cells from different treatment groups. After processing, remove the supernatant, wash the cells with PBS, and then incubate at 37 °C for 20 min in serum‐free medium containing DCFH DA. Then use Hoechst 33 342 for nuclear staining for 10 min. After three additional washes with PBS, intracellular ROS levels were observed and captured using CLSM.

### Mitochondrial Membrane Potential Change

Inoculate 4T1 cells at a density of 1 × 105 cells per room into a 4‐cell confocal culture dish and incubate for 24 h under standard culture conditions to allow cell attachment. Subsequently, the aging group cells were induced to age for 3 days, followed by different treatments, and then incubated for another 3 h. Cells were stained with JC‐1 dye for 30 min and Hoechst 33 342 for 10 min, washed twice with PBS, and imaged using CLSM. Analyze changes in mitochondrial membrane potential by comparing the ratio of red and green fluorescence to reflect cellular damage.

### Western Blot Analysis of Senescence Pathway and HSP70

For HSP70, the density of 2 × 10^5^ 4T1 cells was spread into 6‐well plates. Before incubation, divide the cells into the following groups: N, S 42 °C, Qu 42 °C, N 42 °C, S + Qu 42 °C, S + IR + L, S + IQ + L. First, the S group cells were induced to senescence with 2.5 µM pal for 3 days, followed by incubation with Qu, IR, and IQ NPs for 8 h. Then, the cells were irradiated with 1.0 W cm^−2^ for 5 min and incubated for another 2 h, the cells were gently collected with a cell scraper and lysed on ice for 30 min by adding a certain amount of lysis solution. The supernatant was centrifuged at 4 °C and 14 400 rpm for 15 min. The protein concentration was then determined using the BCA Protein Quantification Kit (Thermo Fisher Scientific (China) Co. Proteins were separated from sodium dodecyl sulfate polyacrylamide gel electrophoresis (SDS‐PAGE) gels. It was then transferred to a polyvinylidene difluoride (PVDF) membrane. The PVDF membrane was then closed with 5% skimmed milk for 2 h at room temperature, washed 5 times with TBST and incubated with primary antibody overnight in a shaker at 4 °C. The membrane was then washed and incubated with secondary antibody at room temperature in a shaker for 2 h. The western blot analysis of senescence pathway is consistent with HSP70. Finally, the protein bands of senescence pathway and HSP70 were visualized by chemiluminescence imaging system (Bio Rad) and quantified by Image J.

### Penetration Ability in 3D Multicellular Spheroids

For 3D multicellular spheroids culture, 4T1 cells were suspended in DMED medium containing 0.1% carboxymethyl cellulose at a density of 2.5 × 10^5^ cells. Place each drop of 25 µL cell suspension on a culture dish cover and invert to culture each tumor spheroids for ≈5–8 days. Then, treat the spheroids with IQ NPs (containing cy5.5 dye) for 8 h. After coincubation for 8 h, the spheroid was exposed under a 1060 nm laser (1 W cm^−2^) for 10 min until destroyed. Then washing the spheroids three times with PBS and perform layer scanning of the tumor spheroids at 20 µm using confocal microscopy (LEICA DMI8).

### Animal Model

All experimental animal procedures were performed with approval from the Institutional Animal Care and Use Committee of the Hospital of Zhejiang Province (GB/T 35892‐2018, 20 240 311 191 710 952 868). Female nude mice aged 4–6 weeks were purchased from Qizhen Experimental Animal Technology Co., Ltd. (Hangzhou, China). To develop subcutaneous tumors, 1 × 10^6^ 4T1 cells suspended in 200 µL PBS were subcutaneously injected into the back of 4‐ to 5‐7‐week‐old female nude mice. In accordance with international animal ethics guidelines, tumor burden must not exceed 10% of body weight, with no single dimension surpassing 20 mm in mice (40 mm in rats). The experiment must be terminated with euthanasia if tumors exhibit ulceration, necrosis, infection, metastasis, rapid growth, or interference with normal functions like feeding or mobility.

### In Vivo Anti‐Tumor Effect

Nude mice inoculated with 4T1 tumors were randomly divided into 7 groups (n = 5). When the tumors grew to ≈100 mm^3^, the 7 groups of mice were divided into N, S, S + Qu, IQ+L, S + IR ± L, and S + IQ ± L groups. First, the S, S + Qu, S + IR ± L, and S + IQ ± L groups mice were orally administered with pal (100 mg kg^−1^) for 7 days, and then the remaining drugs were given to the mice. Record real‐time temperature changes at the tumor site of mice during laser irradiation (every 2 min for a total of 5 times). Record the size of the tumor and the weight of the mice every 2 days for each group. On the 14th day, dissection was performed on all mice to take tumors and organs (heart, liver, spleen, lung, and kidney), then H&E, HSP70, HSP90 and Ki67, staining was used to analyze tissue damage and thermal resistance capacity after different treatments. And remove the serum from each group of mice to evaluate the in vivo toxicity of IQ NPs.

### Hemolysis Test

Prepare a series of IQ NPs under the concentration gradient (0, 25, 50, 75, and 100 µg mL^−1^), and transfer them into EP tubes containing 1 ml of physiological saline (dilute IQ NPs into corresponding concentrations with 1 ml of physiological saline. Plus an additional group of ddH_2_O alone as positive group, making a total of 6 groups). Then, add 20 µL of mouse red blood cells (RBC) into each centrifuge tube (collect whole blood with an anticoagulant tube, take the whole blood of mice, centrifuge at 3000 rpm for 15 min at room temperature. Discard the supernatant serum to obtain the RBC suspension). After incubating the 6 groups at 37 °C for 2 h, spin them at 2000 rpm for 5 min. Then collect the supernatant and measure the absorbance as OD value at 545 nm. Set the ddH_2_O group as the positive group and the saline group as the negative group. Calculate the hemolysis rate using the following formula:

(4) $$Hemolysis\;rate\;\;\left(\%\right)=\frac{OD_{sample}-OD_{negative}}{OD_{positive}-OD_{negative}}\times 100\%$$

*OD_sample_* refers to the ultraviolet absorption of the sample supernatant at 545 nm; *OD_negative_* refers to the ultraviolet absorption of the saline supernatant at 545 nm; *OD_positive_* refers to the ultraviolet absorption of the ddH_2_O supernatant at 545 nm.

Unless otherwise stated, experimental measurements were taken in at least triplicate. Results are expressed as mean ± standard deviation (Mean ± S.D.). Statistical significance is expressed as *p < 0.05, **p < 0.01, ***p < 0.001, ****p < 0.0001.

### Semi‐Quantification by Image J

For each experiment, three or more fields of view were taken as fluorescent images for each group. The fluorescent intensity in each image was semi‐quantitated with Image J and averaged. Results were displayed as mean fluorescence intensity.

### Statistical Analysis

All statistical analyses were performed using GraphPad Prism 9.5. Unless otherwise stated, experimental measurements were taken in at least triplicate. Results were expressed as mean ± SD. Statistical differences between the two groups were tested using the two‐way ANOVA test with Tukey's/Šidák's post hoc tests (α = 0.05, assumptions verified). Statistical significance was expressed as *p < 0.05, **p < 0.001, ***p < 0.0001, ****p < 0.00001. Figure legends specify n, tests, data presentation, and p values.

## Conflict of Interest

The authors declare no conflict of interest.

## Supporting information



Supporting Information

## Data Availability

The data that support the findings of this study are available from the corresponding author upon reasonable request.
