# Supplementary material for: Senescence‐Inducing Therapy Sequential NIR‐II Mild Photothermal/Senolytic Therapy of Triple Negative Breast Cancer
Source: Adv Sci (Weinh). 2025 Aug 13;12(42):e07248. doi: 10.1002/advs.202507248 (PMC12622523; doi:10.1002/advs.202507248)
Supplement: Supplementary file 1 — Supporting Information [file ADVS-12-e07248-s001.docx]

**Senescence-Inducing Therapy** **Sequential NIR-II** **Mild Photothermal/Senolytic Therapy of Triple Negative Breast Cancer**

Liya Yu^1^, Yehui Kang^1^, Mengdie Yu^1^, Yuejia Ding^1^, Yang Chen^1^, Qingyuan Wu^2^, Yu Cai^1^*, Huiyu Liu^2^*, Zhenye Lv^1^*

^1^General Surgery, Cancer Center, Department of Breast Surgery, Zhejiang Provincial People's Hospital (Affiliated People's Hospital), Hangzhou Medical College, Hangzhou, Zhejiang, 310014, China.

^2^Beijing Advanced Innovation Center for Soft Matter Science and Engineering, State Key Laboratory of Organic-Inorganic Composites, Beijing Laboratory of Biomedical Materials, Bionanomaterials & Translational Engineering Laboratory, Beijing Key Laboratory of Bioprocess Beijing Laboratory of Biomedical Materials, Beijing University of Chemical Technology, Beijing, 100029, P.R. China.

E-mail: [caiyu@hmc.edu.cn](mailto:caiyu@hmc.edu.cn); [liuhy@mail.buct.edu.cn](mailto:liuhy@mail.buct.edu.cn); lvzhenye@hmc.edu.cn


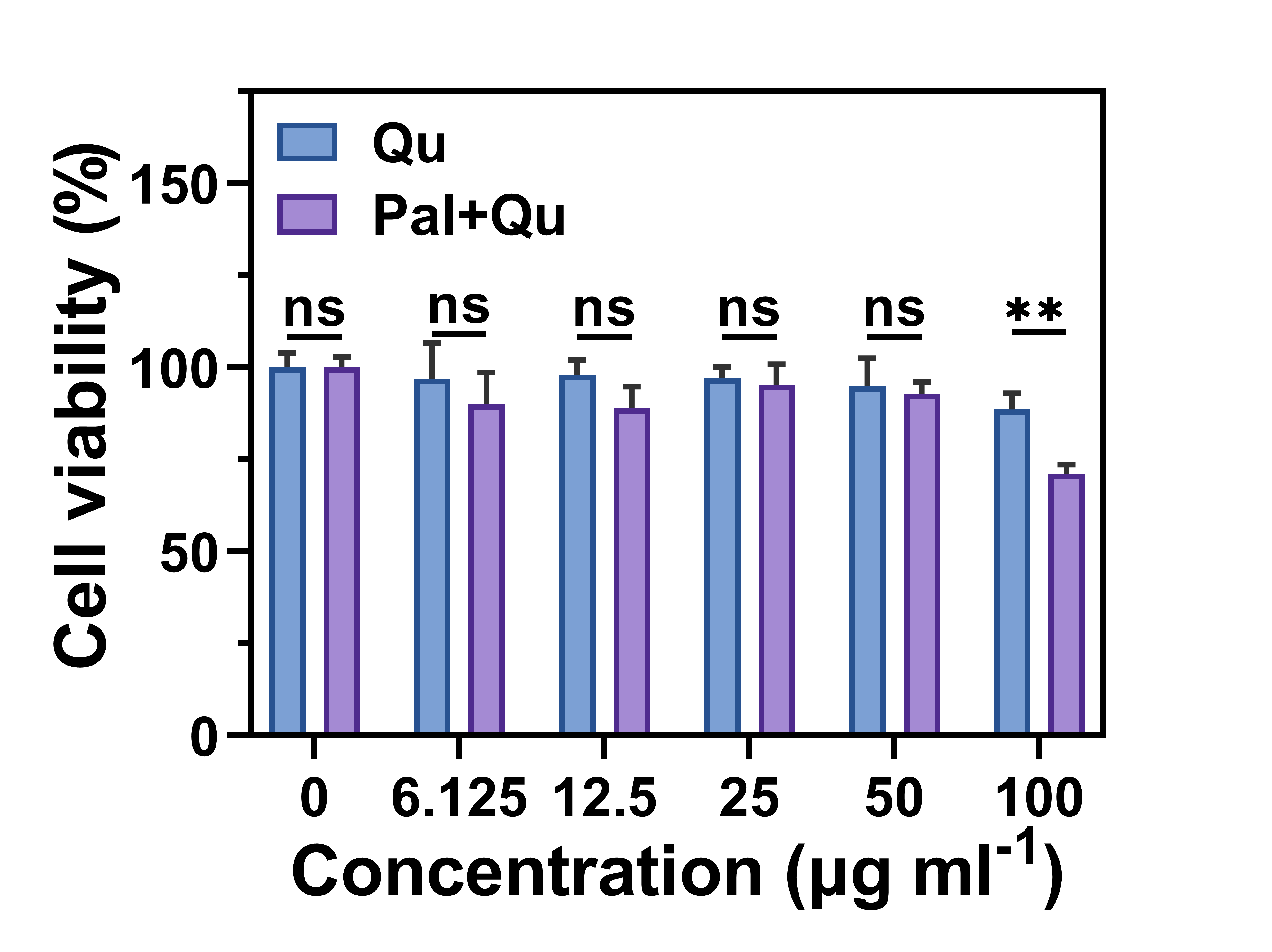


**Figure S1.** Cytotoxicity experiments on Qu and on pal + Qu at different concentrations (Mean ± S.D., *n*=3, two-way ANOVA test, ɑ=0.05, ***p* < 0.001).


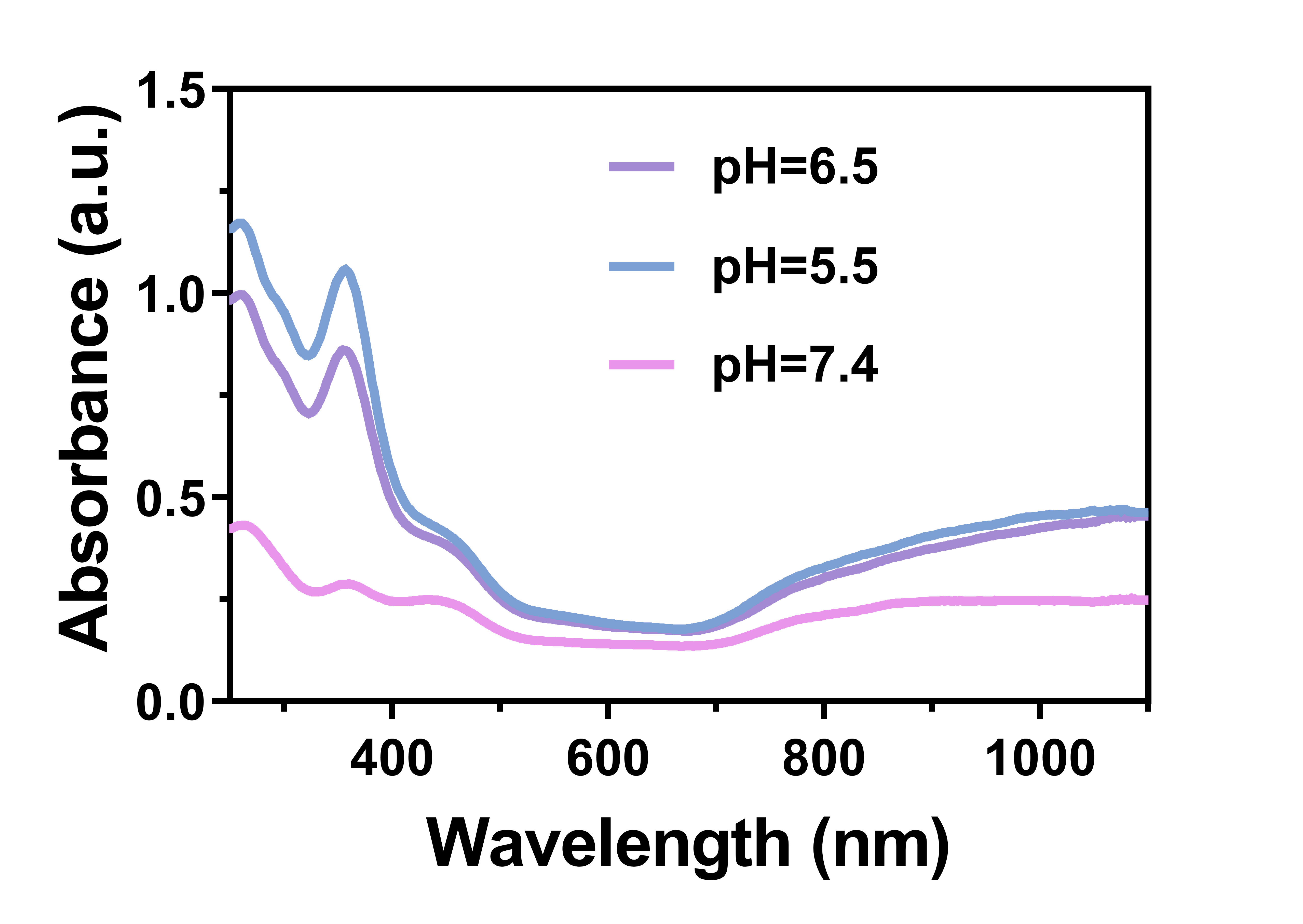


**Figure S2.** The UV-Vis-NIR absorption spectra of IQ NPs in PBS with different pH values.


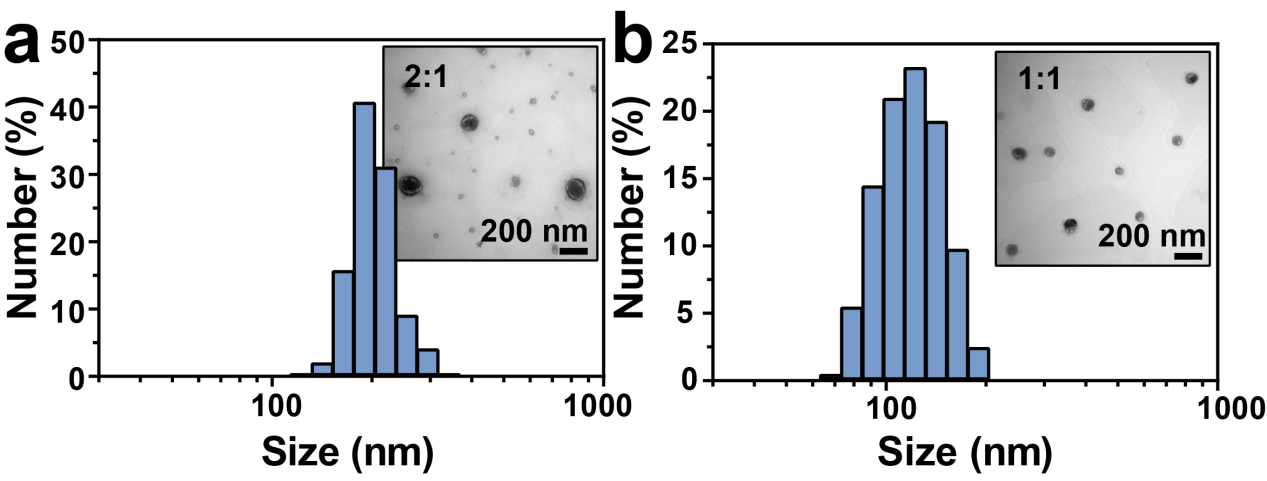


**Figure S3.** TEM and particle size of IR:Qu at different ratios. (a) TEM and particle size of 2:1 IQ NPs. (b) TEM and particle size of 1:1 IQ NPs.


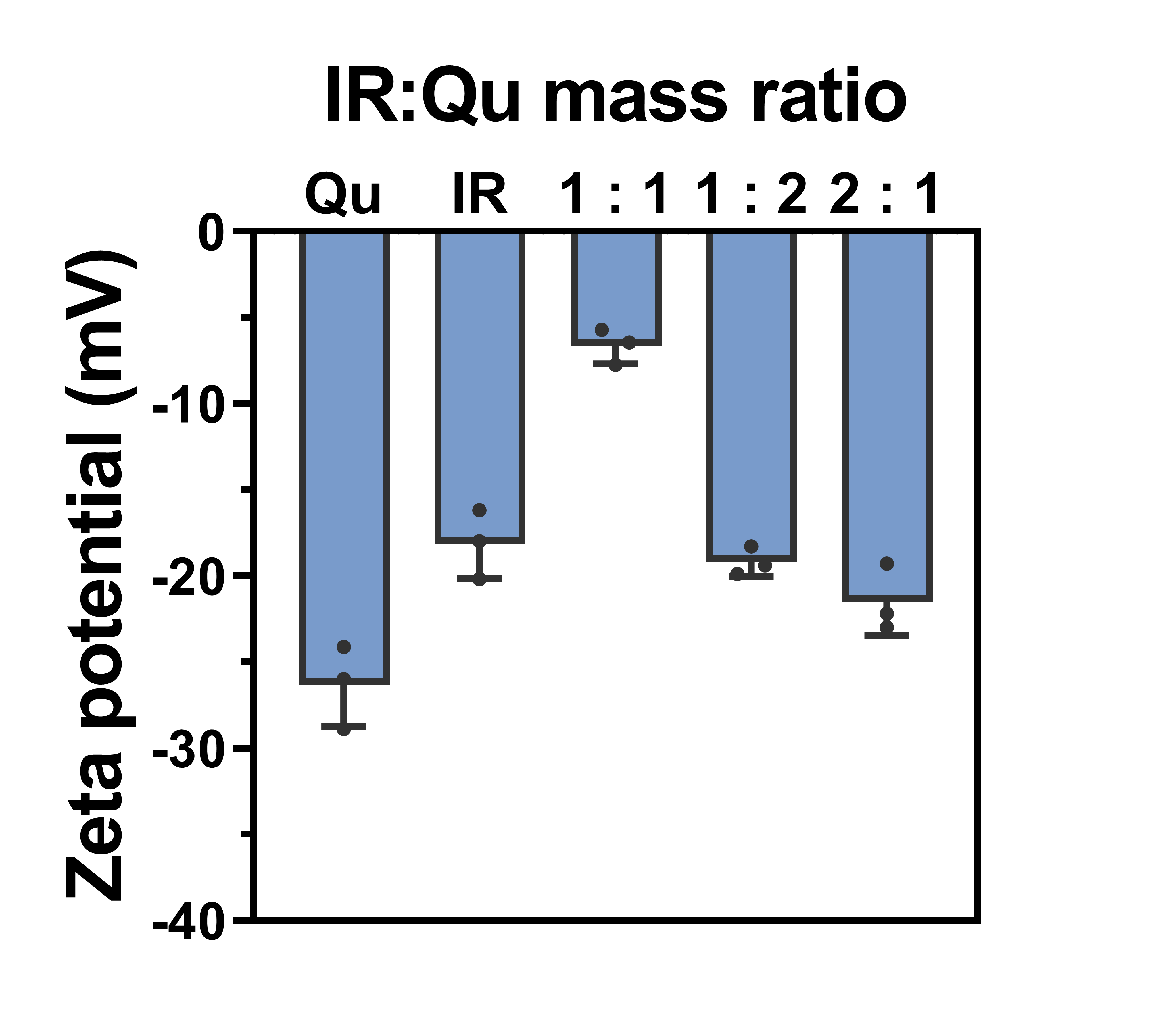


**Figure S4.** Zeta potential of IQ NPs with Qu, IR and different IR to Qu mass ratios.


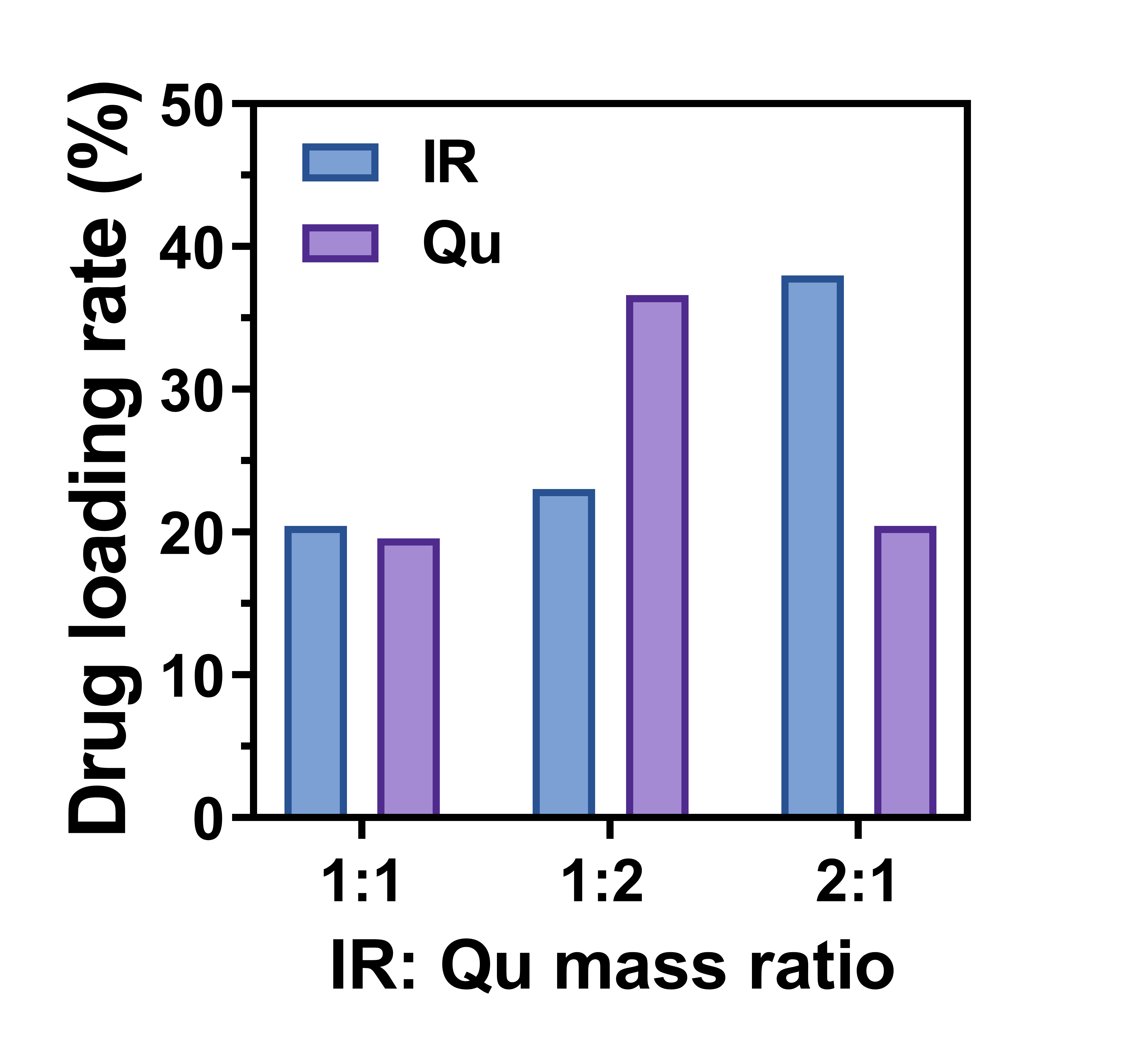


**Figure S5.** The drug loading rate of IQ NPs with different IR and Qu mass ratios.


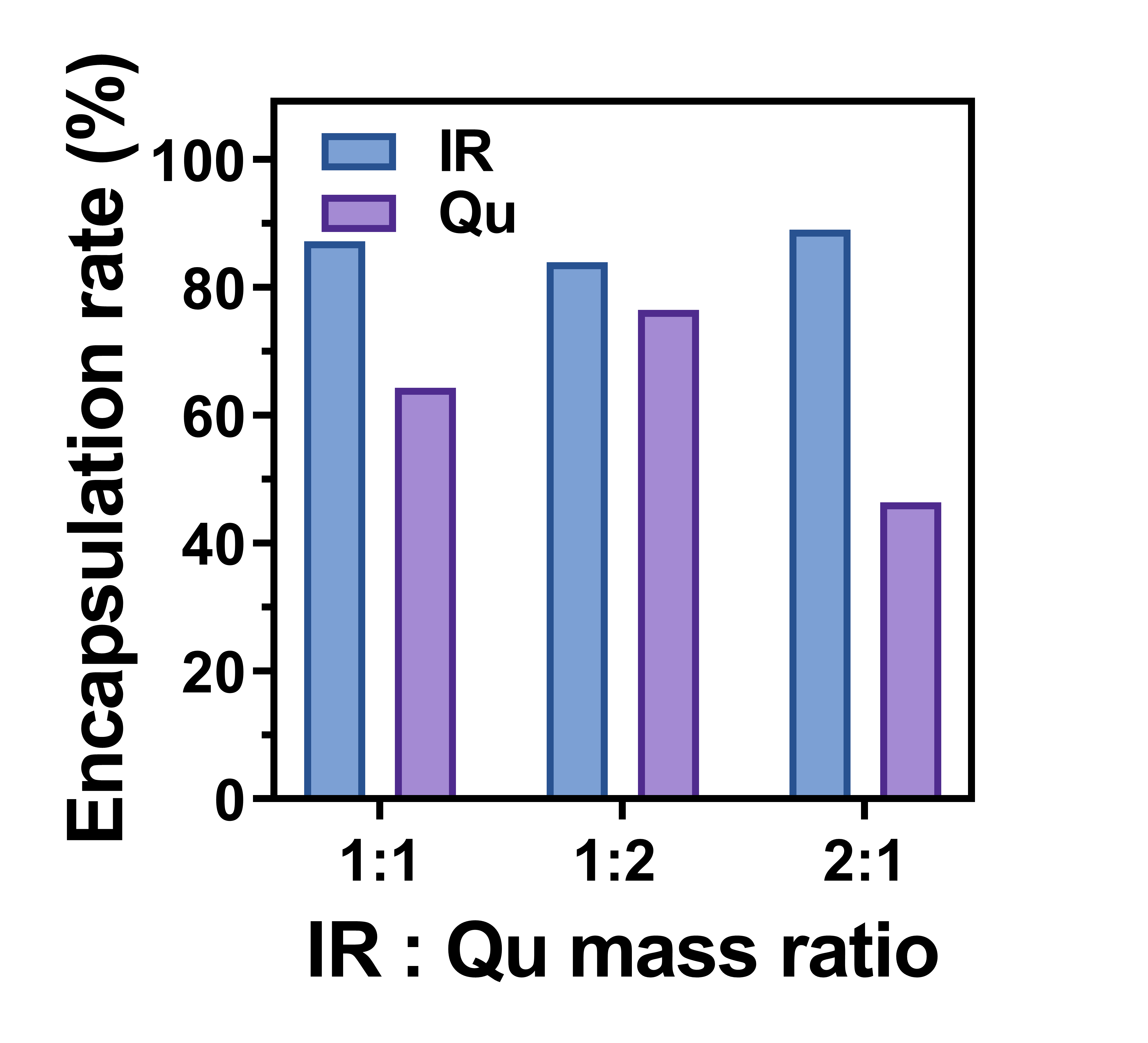


**Figure S6.** The encapsulation efficiency of IQ NPs with different IR and Qu ratios.


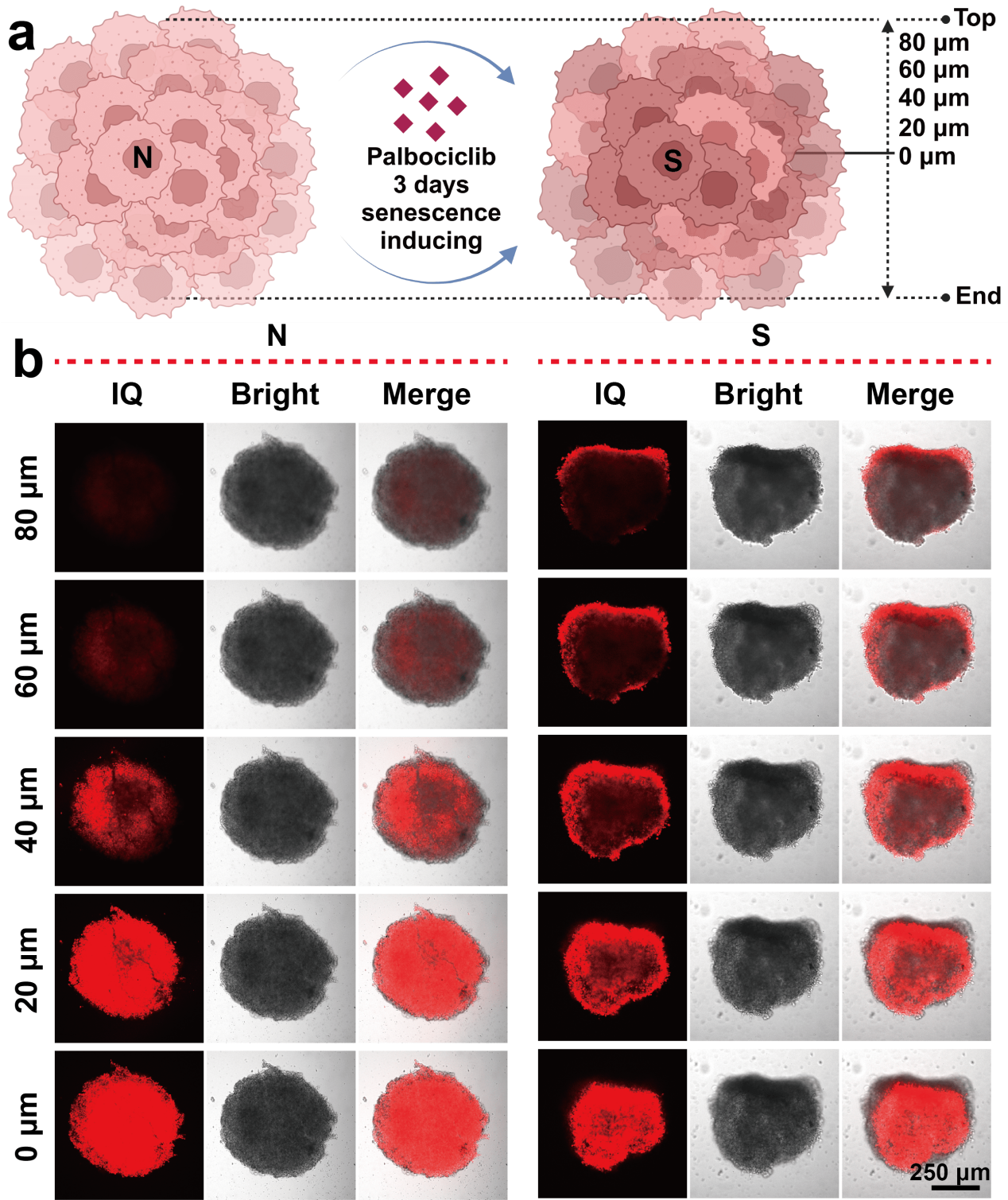


**Figure S7**. Penetration of IQ NPs in solid tumor spheroid model in vitro. (a) schematic diagram of 3D multicellular tumor spheroids. (b) the penetration ability of IQ NPs after co-cultivation for 8 hours.


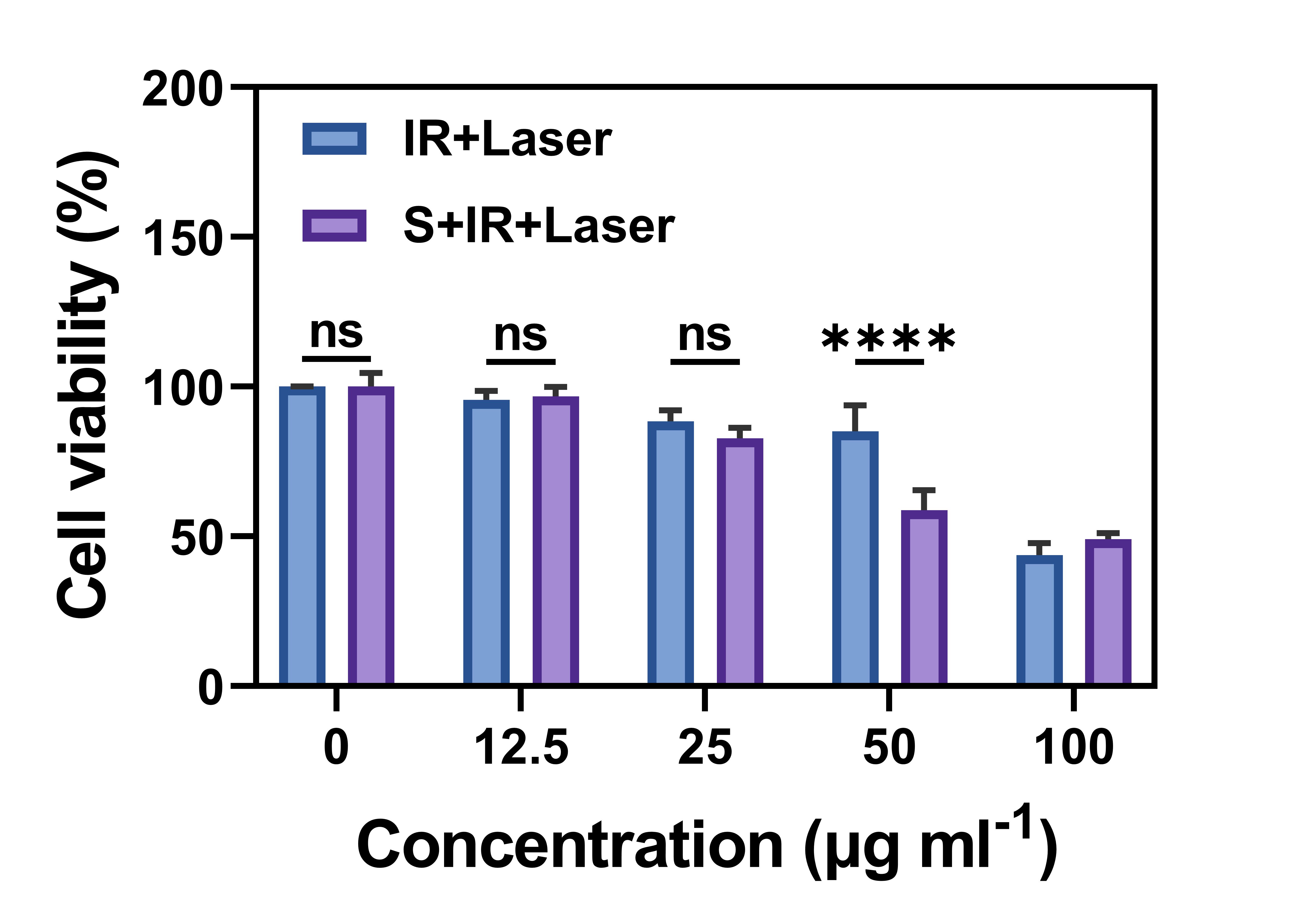


**Figure S8**. test the effect of different concentrations of IR and S+IR on the survival rate with or without laser irradiation (1060 nm, 1.0 W cm^-2^) (Mean ± S.D., n=3, two-way ANOVA test, ɑ=0.05, ****p < 0.00001).


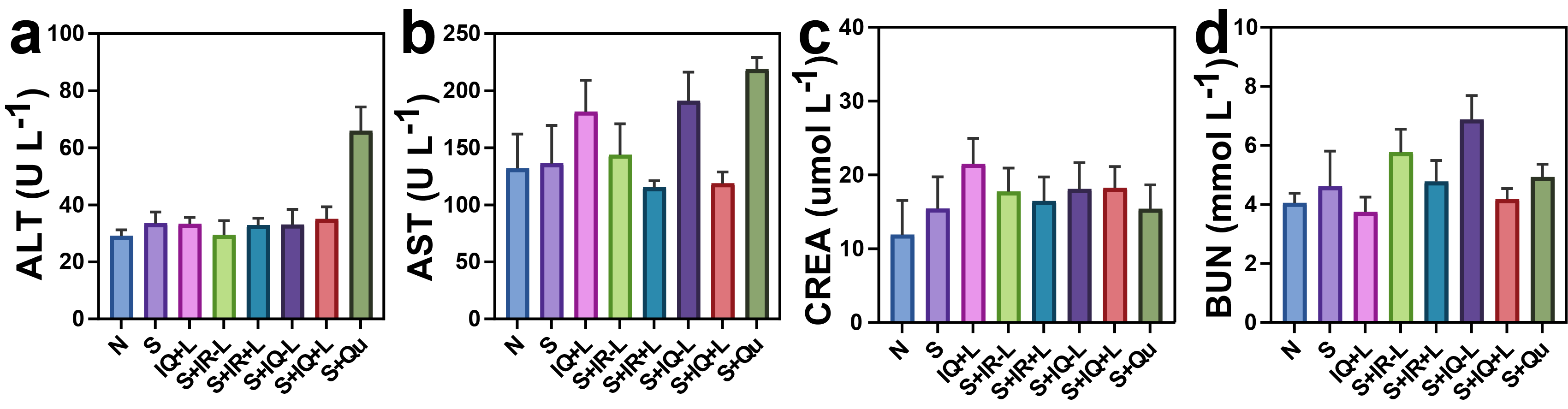


**Figure S9.** Blood routine examinations and blood biochemistry analysis. Blood ALT (Normal value range: 10.06-96.47 U L^-1^), AST (Normal value range: 36.31-235.48 U L^-1^), CREA (Normal value range: 10.91-85.09 μmol L^-1^), BUN (Normal value range: 0-10 mmol L^-1^) biochemistry analysis of various groups after treatments.
